# NS Segment of a 1918 Influenza A Virus-Descendent Enhances Replication of H1N1pdm09 and Virus-Induced Cellular Immune Response in Mammalian and Avian Systems

**DOI:** 10.3389/fmicb.2018.00526

**Published:** 2018-03-22

**Authors:** Henning Petersen, Ahmed Mostafa, Mohamed A. Tantawy, Azeem A. Iqbal, Donata Hoffmann, Aravind Tallam, Balachandar Selvakumar, Frank Pessler, Martin Beer, Silke Rautenschlein, Stephan Pleschka

**Affiliations:** ^1^Clinic for Poultry, University of Veterinary Medicine Hannover, Hanover, Germany; ^2^Institute of Medical Virology, Justus Liebig University Giessen, Giessen, Germany; ^3^Center of Scientific Excellence for Influenza Viruses, National Research Centre (NRC), Cairo, Egypt; ^4^Institute for Experimental Infection Research, TWINCORE Centre for Experimental and Clinical Infection Research, Hanover, Germany; ^5^Department of Hormones, Medical Research Division, National Research Centre, Cairo, Egypt; ^6^Helmholtz Centre for Infection Research, Braunschweig, Germany; ^7^Institute of Diagnostic Virology, Friedrich-Loeffler-Institut, Greifswald, Germany; ^8^Max-Planck Laboratory for Heart and Lung Research, Instituto de Investigación en Biomedicina de Buenos Aires (IBioBA) – CONICET-Partner Institute of the Max Planck Society, Buenos Aires, Argentina

**Keywords:** H1N1pdm09, influenza virus, NS segment, reassortment, innate immunity

## Abstract

The 2009 pandemic influenza A virus (IAV) H1N1 strain (H1N1pdm09) has widely spread and is circulating in humans and swine together with other human and avian IAVs. This fact raises the concern that reassortment between H1N1pdm09 and co-circulating viruses might lead to an increase of H1N1pdm09 pathogenicity in different susceptible host species. Herein, we explored the potential of different NS segments to enhance the replication dynamics, pathogenicity and host range of H1N1pdm09 strain A/Giessen/06/09 (Gi-wt). The NS segments were derived from (i) human H1N1- and H3N2 IAVs, (ii) highly pathogenic- (H5- or H7-subtypes) or (iii) low pathogenic avian influenza viruses (H7- or H9-subtypes). A significant increase of growth kinetics in A549 (human lung epithelia) and NPTr (porcine tracheal epithelia) cells was only noticed *in vitro* for the reassortant Gi-NS-PR8 carrying the NS segment of the 1918-descendent A/Puerto Rico/8/34 (PR8-wt, H1N1), whereas all other reassortants showed either reduced or comparable replication efficiencies. Analysis using *ex vivo* tracheal organ cultures of turkeys (TOC-Tu), a species susceptible to IAV H1N1 infection, demonstrated increased replication of Gi-NS-PR8 compared to Gi-wt. Also, Gi-NS-PR8 induced a markedly higher expression of immunoregulatory and pro-inflammatory cytokines, chemokines and interferon-stimulated genes in A549 cells, THP-1-derived macrophages (dHTP) and TOC-Tu. *In vivo*, Gi-NS-PR8 induced an earlier onset of mortality than Gi-wt in mice, whereas, 6-week-old chickens were found to be resistant to both viruses. These data suggest that the specific characteristics of the PR8 NS segments can impact on replication, virus induced cellular immune responses and pathogenicity of the H1N1pdm09 in different avian and mammalian host species.

## Introduction

Throughout the last century and in the recent past, influenza A viruses (IAVs) have led to drastic outbreaks and pandemics in poultry and humans, respectively (Lai et al., 2016). The most devastating documented outbreak was the pandemic of 1918, which is assumed to have caused 20–50 million casualties worldwide (Morens and Fauci, 2007). IAVs, which crossed the animal/human interface, were likely to have spread silently in birds or other animal reservoirs before (Short et al., 2015). Some IAVs acquired adaptive amino acid (aa) mutations and/or genetically exchanged segments (reassortment) with co-infecting IAV strains, occasionally gaining extended virus receptor binding specificity toward humans and became more virulent causing fatalities and/or pandemics (Morens and Fauci, 2007; Neumann et al., 2009; Pappas et al., 2010; Watanabe et al., 2011; Shi et al., 2013). In 2009, the world was confronted with the first pandemic of this century caused by the swine-originated H1N1-type IAV (H1N1pdm09) (Lessler et al., 2009). This pandemic IAV strain evolved following multiple reassortment events including genomic segments from two swine IAV strains [North American classical swine viruses (HA, NP, and NS) and Eurasian avian-like swine viruses (NA, M)], one human strain [North American human H3N2 viruses (PB1)], and one avian strain [North American avian viruses (PB2, PA)] (Neumann et al., 2009). Surveillance studies revealed multiple zoonotic and anthroponotic transmissions of H1N1pdm09 with an increasing number of circulating lineages and reassortant H1N1pdm09-derived genotypes (Watson et al., 2015). Evidence of H1N1pdm09 in turkey flocks complicates the situation even more, since interspecies transmission of H1N1 IAV between turkeys and swineherds was documented (Wright et al., 1992; Mathieu et al., 2010; Reid et al., 2012). As H1N1pdm09 is circulating in mammals and birds together with seasonal IAVs (H1N1- and H3N2-type), and occasionally with 2013/H7N9- and H5N1-type highly pathogenic avian influenza virus (HPAIV) (Reid et al., 2012; Abdelwhab et al., 2016; Zhu et al., 2016), H1N1pdm09 genome reassortment and evolution, resulting in variants of unknown virulence for mammalian and avian species, is a possible event. Furthermore, gene segments highly related to those of the 1918 pandemic virus can be found in circulating avian IAVs, suggesting that 1918-like variants may emerge in the future (Watanabe et al., 2014).

Segment 8 is the smallest viral genome segment (860–890 nucleotides) that encodes NS1 protein (approximately 230 aa). The spliced mRNA codes for the nuclear export protein NEP/NS2 protein (121 aa) (Lamb and Lai, 1980). Generally, NS1 proteins are grouped into two alleles (A and B) according to their structural homology. Allele A is more common and represents viruses of avian and mammalian origin, while allele B is found exclusively in avian influenza viruses (AIV) (Ludwig et al., 1991). The length of NS1 is variable (202–237 aa) due to occasional internal deletions and/or truncations in the tail region (Hale et al., 2008). In the virion, NS1 is found at very low levels, but it is abundant in the nucleus of IAV-infected cells at an early stage and is later exported into the cytoplasm, where it has diverse functions (Sato et al., 2003; Garaigorta et al., 2005; Melen et al., 2007; Hutchinson et al., 2014). Structurally, NS1 protein comprises two domains, the RNA binding domain (RBD, 1–73 aa) and the effector domain (ED, 88–202 aa), which are connected with the linker region (LR, 74–87 aa) and terminate with the C-terminal tail (CT, 203–237) (Hale, 2014). The RBD contains one or two nuclear localization signals (NLS), allowing active nuclear import and early translocation of NS1 into the nucleus through binding to cellular importin-α (Melen et al., 2007). Moreover, the RBD includes a poly(A) protein (PABPI) binding site that enables binding of NS1 to different RNA species such as viral genomic RNAs, viral mRNAs, poly-adenylated mRNAs, small nuclear RNAs and double stranded RNAs (Marc, 2014). While the ED has specific regions to interact with several host factors including the cleavage and polyadenylation specificity factor 30 (CPSF-30), eukaryotic translation initiation factor 4GI (eIF4GI), polyadenine binding protein II (PABPII), p85b-subunit of phosphatidylinositol 3-kinase (PI3K), and RNA-activated protein kinase R (PKR) (Hale et al., 2008). The NS1 interferes with several signaling pathways and antagonizes antiviral defenses (such as interferon expression) through interactions with RIG-1, TRIM25, PKR, PACT, PI3K, and PDZ-domain containing proteins (Fan et al., 2013; Marc, 2014). In addition, NS1 of IAV contains two NLS to mediate its active nuclear import via binding to host importin-α (Melen et al., 2007; Hale et al., 2008). The NLS1 resides in the N-terminal RBD, whereas the NLS2 is located at the C-terminus disordered tail (Melen et al., 2007). In addition, NS1 protein contains, at its C-terminus, a nucleolar-localization signal (NoLS) (aa 219K, 220R, 224R, and 229K) (Melen et al., 2007). The C-terminal 11-aa truncation (219–230) in NS1 of H1N1pdm09 results in the loss of the NoLS and partially of the NLS2, leading probably to an altered intracellular NS1 localization (Tu et al., 2011).

Some IAV proteins including NS1 contribute to IAV-induced cellular apoptosis (Gannage et al., 2009; Krumbholz et al., 2011; Zhang et al., 2011). Apoptosis is one of the host defense mechanisms minimizing replication of the invading virus during the early stage of infection (Herold et al., 2012). Nevertheless, IAVs also seem to employ apoptosis to facilitate their own replication and pathogenicity (Herold et al., 2012; Muhlbauer et al., 2015). These diverse functions make NS1 protein play a pivotal role in the host/pathogen interaction affecting viral replication efficiency, virus-induced cellular response, host range and pathogenicity of IAV for mammals and poultry, which can be extended further by acquiring certain mutations or by exchange with another NS segments (Ma et al., 2010; Wang et al., 2010; Kanrai et al., 2016).

In order to investigate the *in vitro* and the *in vivo* impact of different NS segments on viral replication kinetics, virus-induced apoptotic effects, virus-induced cellular responses and pathogenicity in mammals and poultry, we used reverse genetics to place the NS segment derived from different IAV subtypes, isolated over 79 years and of alleles A and B (H1N1, 1934, A; H3N2, 1975, A; H5N1, 2004, A; H7N3, 2000, B; H7N7, 1980, A; H7N9, 2013, A; and H9N2, 1998, A) in the genetic background of H1N1pdm09 [A/Giessen/06/2009 (H1N1, Gi-wt)].

## Materials and Methods

### Ethics Approval Statement

All animal trials were conducted in accordance with the recommendations and guidelines of the German Animal Welfare Legislation. The animal trial in chickens was approved by the Committee on the Ethics of Animal Experiments of the Federal State of Mecklenburg- Western Pomerania, Germany (approval number LALLF MV7221.3-1-024/14). The animal trial in mice was approved by the Committee on the Ethics of Animal Experiments of the Federal State of Lower-Saxony, Germany (approval number AZ 33.9-42502-04-12/0939).

### Cells

MDCK-II (Madin-Darby canine kidney cells type II), 293T (human embryonic kidney cells expressing the SV40 large T-antigen), NPTr (newborn pig trachea cells) and A549 (human lung carcinoma cells) were maintained in DMEM (Gibco, Invitrogen) supplemented with 1% Penicillin/Streptomycin (100 IU/ml penicillin, 100 μg/ml streptomycin; Invitrogen) and 10% fetal bovine serum (FBS; PAA Laboratories). Quail-origin (QT-6) fibroblast cells were maintained in Ham’s F-12 medium (Gibco, Invitrogen) containing 1% L-glutamine, supplemented with 8% heat-inactivated FCS, 2% chicken serum, 2% tryptose phosphate broth, and 100 IU penicillin/streptomycin ml^-1^ (Pen/Strep, Gibco, Invitrogen). All cells were incubated at 37°C in the presence of 5% CO_2_. The THP-1 human monocytic leukemia cell line was maintained at 2 × 10^5^ cells/ml in RPMI 1640 medium (Gibco, Invitrogen) supplemented with 10% FBS and 2 mmol/L L-glutamine (Gibco, Invitrogen). THP-1 cells (2 × 10^5^ cells/ml) were differentiated in 24 well plates using 200 nM phorbol 12-myristate 13-acetate (PMA; Sigma-Aldrich) for 3 days. The differentiation of PMA treated cells was enhanced by removing the PMA enriched media, and subsequently incubating the cells in fresh RPMI 1640 (10% FCS, 1% L-glutamine) for another day before infection.

### Construction of Plasmids

A complete set of pMP*ccd*B plasmids encoding the eight viral segments of A/Giessen/06/09 (Gi-wt) virus and the NS segments derived from Influenza A/Victoria/3/75 (H3N2), A/Thailand/KAN-1/2004 (H5N1), A/Anhui/1/2013 (H7N9), A/FPV/Bratislava/79 (H7N7) and A/chicken/Saudi Arabia/CP7/98 (H9N2) were constructed as previously described (Hoffmann et al., 2001; Mostafa et al., 2013). Briefly, the NS segments were amplified by RT-PCR, cloned into pMP*ccd*B (Mostafa et al., 2013), and subsequently used to generate recombinant virus by reverse genetics as previously described (Hoffmann et al., 2000; Mostafa et al., 2013, 2015). The NS segments of A/Mallard/NL/12/2000 (H7N3) and A/Puerto Rico/8/34 (H1N1, PR8-wt) were subcloned from pHH21 or pHW2000, respectively, into pMP*ccd*B (Mostafa et al., 2013).

### Generation of Recombinant/Reassortant Viruses

The Gi-wt and different Gi-NS-reassortants were generated from the eight plasmids reverse genetics system as previously described (Hoffmann et al., 2000; Mostafa et al., 2013) using “Trans-IT2020” for transfection of 293T/MDCK-II co-cultures. The generated viruses were further propagated in MDCK-II cells for 48 h; the supernatants were clarified from cell debris by centrifugation at 2500 rpm for 5 min and then stored in aliquots at -70°C for further experiments. The eight viral segments of the rescued IAVs were verified by sequencing.

### *In Vitro* Replication Kinetics of Gi-NS-Reassortants vs. Gi-wt

To investigate the multistep growth kinetics of recombinant Gi-wt and different Gi-NS-reassortants, A549 and NPTr cells were inoculated with the predefined viruses at a multiplicity of infection (MOI) of 0.001. After 1 h of virus inoculation at RT, cell monolayers were washed with 1x PBS and overlaid with 2 ml of infection medium (Dulbecco’s Modified Eagle Medium (Gibco, Invitrogen) supplemented with 1% Penicillin/Streptomycin, 0.3% BSA and 1 μg/ml of L-(tosylamido-2-phenyl) ethyl chloromethyl ketone (TPCK)-treated trypsin (PAA Laboratories)). Aliquots of 200 μl were collected at 12, 24, 36, and 48 h post infection (p.i.). IAV titration of supernatants was performed by focus forming assay as previously described (Ma et al., 2010).

### Western Blotting Analysis

A549 cells were infected with Gi-wt, Gi-NS-PR8, and Gi-NS-Vict at MOI = 1. At 24 h p.i., the cells were harvested, pelleted and subjected to protein extraction as previously described (Pleschka et al., 2001). A sample (10 μl) of each heated protein extract was then separated on precast gradient NuPAGE^®^ Novex^®^ 4–12% Bis-Tris protein gels (Invitrogen) and subsequently transferred onto immobilon-FL polyvinylidene fluoride (PVDF) membranes (Merck Millipore). Following protein transfer, the PVDF membrane was blocked using blocking buffer [1x TBS (20 mM Tris-HCl, pH 7.6, 140 mM NaCl) containing 5% non-fat dry milk] for 1 h at room temperature (RT). The membrane was washed once for 5 min using washing buffer [1x TBS-Tween (20 mM Tris-HCl, pH 7.6, 140 mM NaCl, 0.05% Tween20)]. Afterward, detection of the viral NS proteins was achieved using mouse monoclonal antibodies recognizing influenza A virus NS1 (Abcam, Cambridge, United Kingdom) diluted 1:2000 in blocking buffer. 1 h later, the membrane was washed three times for 5 min with washing buffer. β-actin was included for normalization and detected using rabbit polyclonal antibody (Abcam) against β-actin diluted 1:10000 in blocking buffer. Next, the membranes were incubated with the corresponding goat anti-mouse IRDye (LI-COR) or goat anti-rabbit IRDye (LI-COR) were diluted 1:10000 in blocking buffer containing a 1:1000 dilution of 10% SDS, in the dark for 1 h. After three washing steps (5 min each), twice with washing buffer and once with 1x TBS, the proteins were visualized using an Odyssey Infrared Imaging System and application software package (LI-COR) and quantified using Quantity One version 4.2.2. Software (Bio-Rad).

### Luciferase Reporter Assay

The Luciferase reporter gene assay was performed as previously described (Hale et al., 2010) with minor modifications. Briefly, 293T cells were seeded in 6-well plates (Greiner; 2 × 10^6^ 293T cells/well) and co-transfected with 1 μg of either pMP-NS-Gi, pMP-NS-PR8, pMP-NS-Vict or empty pHW2000 plasmids mixed with 40 ng pRL-SV40 (Renilla luciferase expression plasmid), and 200 ng p125-Luc (Firefly luciferase plasmid) (Yoneyama et al., 1998), which contains the luciferase reporter gene under the control of the IFN-β promoter. Transfection was performed using Trans-IT2020 as previously described (Mostafa et al., 2013) for 8 h. Except for the mock, transfected cells were stimulated either by infection with virulent Newcastle disease virus (NDV, strain Herts 33/56) at MOI = 3 or treated with DMEM containing 50 ng/ml of recombinant human TNF-α (Invitrogen). After 24 h of stimulation, the cells were harvested, washed one time with 1x PBS, and lysed with 200 μl of “1x passive lysis buffer” (Promega). The amount of Firefly/Renilla luciferase was quantified using the Dual-Luciferase Reporter Assay System (Promega) and measured using a Spark 10M multimode microplate reader (TECAN). Relative luminometer units (RLU), normalized to Renilla luciferase, refer to fold induction of IFN-β promoter activity.

### TUNEL Assay (*In Situ* Cell Death Detection)

The TUNEL assay was performed using the “*In Situ* Cell Death Detection Kit” (Roche, United States), according to the manufacturer’s instructions. Briefly, A549 cells were seeded in 6-well plates (2 × 10^6^/well) overnight. Afterward, cells were infected with reassortant viruses at MOI = 3 for 1 h, incubated with fresh infection media for 10 h at 37°C, washed with 1× PBS, dissociated using Accutase cell dissociation reagent (Invitrogen) and transferred into a 1.5 ml Eppendorf tube. The cells were then fixed with 4% paraformaldehyde (PFA, Sigma-Aldrich) in 1x PBS (pH 7.4) overnight at 4°C, washed twice with 1x PBS and centrifuged at 6000 rpm for 5 min.

The cell pellets were permeabilized with 1% Triton X-100 in 0.1% sodium citrate on ice for 20 min. Cells were washed with 1x PBS and incubated with 50 μl reaction mixture (provided in “*In Situ* Cell Death Detection Kit”, Invitrogen) or the “no enzyme” control in 50 μl label solution (provided in “*In Situ* Cell Death Detection Kit”, Invitrogen) for 1 h in the dark. Finally, cells were washed twice with 1x PBS and fixed with 3.7% formaldehyde in 1x PBS. Fluorescence was quantified by flow cytometry (BD LSRFortessa Cell Analyzer, BD Biosciences).

### Confocal Microscopy

Confluent A549 cells were trypsinized with 1x trypsin-EDTA, reseeded in a 3.5 cm dish (Nunc) containing sterile glass cover-slips (12 mm) and incubated at 37°C with 5% CO_2_. When cells were confluent on the next day, they were infected with reassortant viruses at MOI = 1. Growth medium was removed from the culture dish at the indicated time points, the cells were washed once with 1x PBS^++^ (PBS containing 100 mg/l CaCL_2_ and 100 mg/l MgCl_2_) and subsequently fixed overnight with 1 ml 4% PFA in 1x PBS^++^ at 4°C. After fixation, cells were washed twice with 1x PBS^++^ and incubated with 1 ml 1% Triton X-100 (in 1x PBS) for 45 min. Subsequently, cells were washed three times with 1x PBS and incubated with 20 μl of the primary mouse anti-Flu A NP mouse antibody (clone 1331, Bio-Rad) [1:200 dilution in PBS^++^/3% bovine serum albumin (BSA)] for 1 h at RT. Afterward, cells were washed three times and incubated with 20 μl of the secondary Alexa 594 goat anti-mouse IgG (1:200 diluted in PBS^++^/3% BSA, Thermo Fisher Scientific) for 1 h at room temperature (RT). The cells were then washed again three times and incubated with 20 μl DAPI (10 mg/ml in PBS++/3% BSA) for 5 min. The cells were subjected to three washes with 1x PBS and an additional wash with ddH_2_O. Finally, cells were washed in PBS and water, embedded in 0.13 M Tris-HCl (Roth) containing 9.1% Mowiol (Sigma-Aldrich), 22.7% glycerol, and 2.5% DABCO (1,4-diazabicyclo[2.2.2] octane; Merck), and visualized using a confocal laser-scanning microscope (Leica TCS SP5, Leica).

### RT-qPCR Analysis of A549 and dTHP-1 Cells

Relative expression of selected mRNAs was quantified following infection of A549 cells and differentiated THP-1 (dTHP-1) with Gi-wt or Gi-NS-PR8. A549 cells were grown in DMEM medium (GIBCO^®^ Life Technologies^TM^) and plated in 24-well plates at a density of 4 × 10^5^ cells per well for the infection experiments. The dTHP-1 cells were grown in RPMI medium (GIBCO^®^ Life Technologies^TM^) and were plated into 24-well plates at a density of 4 × 10^5^ cells per well, where they were differentiated as described above. Infections of A549 and dTHP-1 cells were carried out at MOI = 1, and the cells were collected in RA1+β-mercaptoethanol lysis buffer (Macherey Nagel) at the indicated time points p.i.. RNA was purified using the Nucleospin RNA purification kit (Macherey Nagel) including on-column removal of DNA by digestion with rDNase (Macherey Nagel) for 15 min. at RT. cDNA was synthesized with the PrimeScript^TM^ kit (TaKaRa) using 400 ng RNA in a 10 μl reaction. RT-qPCR reactions were set up in a final volume of 20 μl, using the SensiFast^TM^ SYBR^®^ No-ROX Kit (Bioline, Taunton, MA, United States) and the primers listed in **Table 1**. RT-qPCR was performed in a LightCycler^®^ 2.0 instrument (Roche), using 45 cycles of the following program: 95°C for 15 s, 60°C for 15 s, and 72°C for 15 s. To exclude artifacts resulting from primer dimer formation, melting curve analysis was performed using the sequence 95°C for 15 s, 60°C for 15 s, 95°C for 1 min and 37°C for 30 s The results correspond to two independent experiments, each containing three biological replicates per condition, amounting to a total of six replicates per condition. Relative expression of the mRNA targets was calculated using the 2^-ΔΔC_T_^ method (Livak and Schmittgen, 2001). Statistical significance was determined with two-way ANOVA and *T*-test.

**Table 1:** Primers used for RT-qPCR analysis of A549 and dTHP-1 cell infections.

Gene	Name	Sequence (5′–3′)	Reference
HA	Pan-H1-F	CTCGTGCTATGGGGCATTCA	Mostafa et al., 2016
	Pan-H1-R	TTGCAATCGTGGACTGGTGT	
β-Actin	β-Actin -F	CATGAAGTGTGACGTGGACATCC	Mukherjee et al., 2011
	β-Actin-R	GCTGATCCACATCTGCTGGAAGG	
IFN-α	IFN-α-F	CTTGTGCCTGGGAGGTTGTC	^∗^
	IFN-α-R	TAGCAGGGGTGAGAGTCTTTG	
IFN-β	IFN-β-F	CAGCAATTTTCAGTGTCAGAAGC	Jin et al., 2010
	IFN-β-R	TCATCCTGTCCTTGAGGCAGT	
IL-6	IL-6-F	ACCTGAACCTTCCAAAGATG	^∗^
	IL-6-R	GCTTGTTCCTCACTACTCTC	
Mx1	MX1_fwd2	ACAGGACCATCGGAATCTTG	Kapadia et al., 2003
	MX1_rev2	CCCTTCTTCAGGTGGAACAC	
IFIT1	IFIT1_rev	GCAGAACGGCTGCCTAATTT	Li et al., 2015
	IFIT1_fwd	TCAGGCATTTCATCGTCATC	
OAS1	OAS1_F	TGACTGGCGGCTATAAACC	^∗^
	OAS1_R	TGGGCTGTGTTGAAATGTGT	
CXCL5	CCL5-F	TACCATGAAGGTCTCCGC	Chakrabarti et al., 2010
	CCL5-R	GACAAAGACGACTGCTGG	
CXCL-10	CxCL-10_F	CTGCTTTGGGGTTTATCAGA	Bibert et al., 2013
	CxCL-10_R	CCACTGAAAGAATTTGGGC	
TNF-α	TNFhu-fw	ACCCTCTCTCCCCTGGAAAGGACA	^∗^
	TNFhu-re	TGAGGAACAAGCACCGCCTGGA	

^∗^*Not previously published*.

### Infection of Mice

Female C57BL/6J mice between 10 and 11 weeks of age were anesthetized by intra-peritoneal injection of Ketamine-Rompun (10 μl/g body weight). IAV Gi-wt and Gi-NS-PR8 were administered intranasally in a total volume of 25 μl sterile PBS at an infectious dose of 5 × 10^5^ FFU/animal (Lv et al., 2014). Mice were kept under specific pathogen free (SPF) conditions. Body weight was monitored daily. Mice showing a weight loss of more than 20% of the starting body weight were euthanized for animal welfare reasons and recorded as dead.

### Infection of QT6 Cells and Avian Tracheal Organ Cultures (TOC)

Multistep replication kinetics was done by inoculating QT6 cells with Gi-wt and Gi-NS-PR8 viruses at a MOI equals 0.001, and incubating for 1 h at RT. Then, cell monolayers were washed with 1x PBS and overlaid with 2 ml of infection Hams-F12 medium, supplemented with 1% Penicillin/Streptomycin, 0.3% BSA and 1 μg/ml of L-(tosylamido-2-phenyl) ethyl chloromethyl ketone (TPCK)-treated trypsin (PAA Laboratories). Aliquots of 200 μl were collected at 12, 24, 36, and 48 h post infection (p.i.). Supernatants were further titrated for progeny virions by focus forming assay as previously described (Ma et al., 2010).

Tracheal organ cultures (TOCs) from turkey were prepared as previously described (Petersen et al., 2012). Briefly, embryonated turkey eggs (Kartzfehn, Boesel) were sacrificed at embryonation day 26. Tracheae were excised and cut into rings of 0.8 – 1.0 mm width. Tracheal rings were individually cultured in maintenance media [Medium 199 + 1% Penicillin/Streptomycin (100 IU/ml penicillin, 100 μg/ml streptomycin; Biochrom)] in an overhead shaker at 37.5°C. Percentage of cilia activity of TOCs was analyzed semi-quantitatively by inverted microscopy. Only TOCs with 100% cilia activity were used for the experiments.

For IAV infection, TOCs were washed with PBS and subsequently inoculated with 10^3^ FFU of wt or different recombinant reassortant IAV in 100 μl PBS/BSA [PBS++ containing 0.2% bovine serum albumin (BSA, PAA)], or with PBS/BSA alone as control, for 1 h at RT. After removal of the inoculum, TOCs were washed with PBS++ and subsequently cultured in 800 μl challenge medium (Medium 199 containing 0.2% BSA) in the overhead shaker at 37.5°C. TOCs were analyzed for cilia activity at 8, 16, 24, 32, and 48 h; supernatants were collected at the same time points and the IAV titer was determined by focus forming assay as previously described (*n* = 6/time point) (Ma et al., 2010). Differences in means between virus titers were tested for significance by ANOVA (Randomized Complete Block Design; Statistix 10, Analytical Software, Tallahassee, FL, United States).

Tracheal organ cultures were individually harvested and homogenized in 500 μl “peqGOLD TriFast” (Precellys 24 homogenizer with CK14 ceramic beads, Peqlab). RNA was extracted and purified according to the manufacturer’s instructions (Peqlab). TaqMan real-time RT-PCR (RT-qPCR) was used to quantify cytokine and ISG mRNA expression in TOCs (*n* = 6/time point/group). Gene specific primers and probes (**Table 2**) were used with the “AgPath-ID One-Step RT-PCR Kit” (Applied Biosystems, Foster City, CA, United States) according to the manufacturer’s instructions. Individual samples were normalized to 28S rRNA expression. Five μl of diluted total RNA were used per 25 μl reactions. RT-qPCR was performed using the Stratagene MX 3005P RT-qPCR detection system (Stratagene, La Jolla, CA, United States) with the following cycle profile: one cycle at 45°C for 10 min and 95°C for 10 min, and 40 cycles of 95°C for 15 s and 57°C for 45 s. For quantification, cycle threshold (*C*_t_) values of expressed mRNA were normalized against the *C*_t_ values of 28S rRNA of the same sample (Δ*C*_t_) as described by Powell et al. (2009). The data are presented as mRNA fold change in relation to Δ*C*_t_ values from non-infected groups. Significant differences between groups were determined by Wilcoxon Rank-Sum Test.

**Table 2:** Primers used for RT-qPCR analysis of infection of turkey tissue.

Gene	Name	Sequence (5′–3′)	Reference
28S	28S F	GGCGAAGCCAGAGGAAACT	Kaiser et al., 2000
	28S R	GACGACCGATTTGCACGTC	
	28S P	(HEX)-AGGACCGCTACGGACCTCCACCA-(TAMRA)	
Turkey IFN-α	TuIFN-α-F	GACAGCCAACGCCAAAGC	Petersen et al., 2013
	TuIFN-α-R	GTGGCTGTCCGCCAAGCATT	
	TuIFN-α-P	(FAM)-CTCAACCAGATCCAGCGGTACGCC-(TAMRA)	
Turkey IFN-β	TuIFN-β-F	CCTCCAACACCTCTTCAACATC	Sid et al., 2016
	TuIFN-β-R	TGGTGTGCGTGGTCAAT	
	TuIFN-β-P	(FAM)-TTAGCAGCCCACACACTCCAGCACACTG-(TAMRA)	
Turkey MX	TuMX-F	CTCAGAGGTGAAGGAAGCAATA	^∗^
	TuMX-R	GGGACCAGATTTCAAGGGAA	
	TuMX-P	(FAM)-AAGCCCAAGATATAGTGGCTGGCA-(TAMRA)	

^∗^*Not previously published*.

### Infection of Chickens

The chicken *in vivo* challenge experiment was conducted in biosafety level 3 containment facilities at the Friedrich-Loeffler-Institut (FLI), Greifswald-Insel Riems, Germany. Ten 6-week-old chickens (VALO SPF; Lohmann Tierzucht GmbH, Cuxhaven) were inoculated oculo-oronasally with 10^6^ 50% tissue culture infectious dose 50 (TCID_50_) of either Gi-wt or Gi-NS-PR8 IAV in 100 μl PBS. Three control birds were inoculated with PBS only. All three chicken groups were housed in different cages in the same room with unlimited access to feed and water. At 24 h post inoculation, three sentinel chickens were housed with each virus-inoculated group to detect possible transmission of viruses. Chickens were controlled daily for clinical signs such as apathy and dyspnea. For the detection of virus shedding, oropharyngeal, and cloacal swabs were collected at 1–7 days post inoculation. RNA was isolated and IAV load of individual samples (single technical replicate) were analyzed by real-time quantitative PCR (RT-qPCR) with the “AgPath-ID One-Step RT-PCR Kit” (Applied Biosystems, Foster City, CA, United States) specifically detecting the hemagglutinin gene of H1N1pdm combined with an internal control system in a duplex assay as described previously (Hoffmann et al., 2010). Blood sampling for the analysis of IAV NP specific antibodies was performed at days 7, 14, and 21 post inoculation. Serum samples from chickens were heat inactivated at 56°C for 30 min and analyzed by means of a commercial enzyme-linked immunosorbent assay (ELISA) for the presence or absence of antibodies against IAV nucleoprotein (NP) (ID Screen^®^ Influenza A Antibody Competition ELISA kit, ID-vet, Montpellier, France) according to the manufacturer’s instruction.

### Biosafety

All experiments with infectious virus were performed according to German regulations for the propagation of influenza viruses. All experiments involving low pathogenic and highly pathogenic avian influenza A viruses were performed in biosafety levels 2 and 3 (BSL3) containment laboratories, respectively, approved for such use by the local authorities.

## Results

### Growth Kinetics of NS-Reassortants of Gi-wt in Mammalian Cells

**FIGURE 1:**
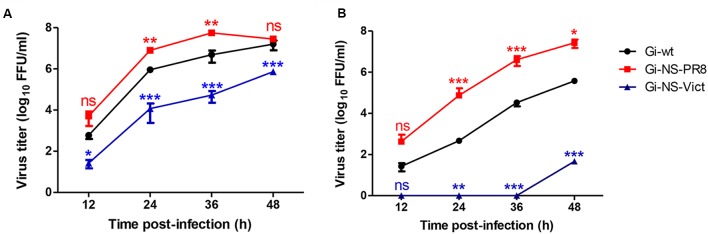
Replication kinetics of Gi-NS-PR8 and Gi-NS-Vict in comparison to recombinant Gi-wt. Human A549 **(A)** and swine NPTr **(B)** cells were inoculated with different Gi-reassortants and recombinant Gi-wt at an MOI of 0.001 Supernatants were collected at 12, 24, 36, and 48 h p.i.. Viral titers were then determined in harvested samples by foci assay (FFU/ml) in MDCK-II cells. The titers were calculated for three independent experiments. Statistical analysis was performed using “repeated measures ANOVA,” followed by “Bonferroni *post hoc*” test. ^∗^*p* < 0.05, ^∗∗^*p* < 0.001, ^∗∗∗^*p* < 0.0001; ns, non-significant. Error bars represent standard deviation (SD).

In order to investigate whether the NS segment of other IAV strains would improve propagation of H1N1pdm09 and/or expand the host range we analyzed the impact of NS reassortment between the pandemic H1N1pdm09 (A/Giessen/06/09, Gi-wt) and other IAVs (HPAIV, LPAIV) on its replication efficiency in human and porcine cells. These recombinant NS reassortants were compared to recombinant Gi-wt in A549 and NPTr cells, respectively. Among the assayed viruses, the Gi-reassortant bearing the NS segment of PR8 (Gi-NS-PR8) showed significant higher replication efficiency compared to Gi-wt at 24 and 36 h post infection (p.i.) in A549 (**Figure 1A**) and NPTr (**Figure 1B**) cells, respectively (*P* < 0.05). In contrast, the Gi-reassortant with the NS-segment from Victoria/H3N2 (Gi-NS-Vict) showed a significant lower replication efficiency compared to recombinant Gi-wt at 24, 36, and 48 h p.i. in both cell lines (**Figures 1A,B**) (*P* < 0.05). The other Gi-reassortants carrying the NS segment of KanI/H5N1 (Gi-NS-KanI), Ma/H7N3 (Gi-NS-Ma), Brat/H7N7 (Gi-NS-Brat), Anhui/H7N9 (Gi-NS-Anhui), and SA/H9N2 (Gi-NS-SA) showed no significant difference in viral replication efficiencies in A549 cells (Supplementary Figure S1), when compared to the Gi-wt (12–48 h p.i.). In NPTr cells, the replication efficiency of these other Gi-reassortants was comparable to Gi-wt at early time points (12–24 h p.i.). At later time points (36 and 48 h p.i.), Gi-wt always showed a significantly higher replication efficiency compared to these Gi-reassortants (Supplementary Figure S1). Thus, only the NS segment of PR8 enhanced the replication efficiency of the reassortant Gi-virus, while the NS segment of Victoria/H3N2 interfered with the replication efficiency of the reassortant Gi-virus and the other NS segments either did not affect or even reduced replication efficiencies of H1N1pdm09.

### Apoptotic Activity and Nuclear RNP Export of Gi-NS-PR8 vs. Gi-wt

**FIGURE 2:**
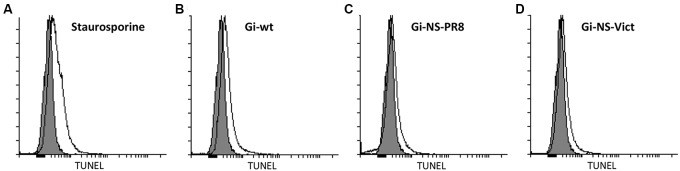
Impact on virus-induced apoptosis by different NS reassortants. A549 cells were infected with recombinant Gi-wt and NS-reassortants of Gi-wt at an MOI of 3. After 10 h, the cells were fixed and permeabilized. The degraded DNA, a marker of apoptosis, was then labeled and quantified using the TUNEL assay. Staurosporine was used as a positive control and “mock” represents the untreated cells (gray histogram). **(A)** Staurosporin-treated A549 cells. **(B)** Gi-wt-infected A549 cells. **(C)** Gi-NS-PR8-infected A549 cells. **(D)** Gi-NS-Vic-infected A549 cells.

Since different studies have reported that H1N1pdm09 can induce apoptosis in late, but not in early stage of viral replication (Yang et al., 2011, 2012), we assessed the ability of the NS reassortants Gi-NS-PR8 and Gi-NS-Vict to induce late stage apoptosis (10 h p.i.) in A549 cells compared to Gi-wt. To this end, cells were infected with recombinant and reassortant viruses at an MOI of 3 and incubated for 10 h. The amount of apoptotic cells was then quantified by FACS analysis. Staurosporine, an inducer of apoptosis, was used as positive control (**Figure 2A**). Similar to Gi-wt (**Figure 2B**), both Gi-NS-PR8 (**Figure 2C**) and Gi-NS-Vict (**Figure 2D**) reassortants showed limited apoptotic activities, at 10 h p.i.. These results revealed that the NS segments of Gi-NS-PR8 and Gi-NS-Vict did not alter the apoptotic potential of the pandemic 2009 Gi-strain. Furthermore, it suggests that the change in replication efficiencies of Gi-NS-PR8 (increased) and Gi-NS-Vict (decreased) was not related to a change of the apoptotic potential of these two Gi-reassortants compared to the recombinant Gi-wt.

We then analyzed the possible effect of the PR8 NEP/NS2 of the Gi-NS-PR8 reassortant virus on nuclear export of viral RNPs as a measure of efficient progeny virion production. Overall, we found an increased expression signal for NP representing the viral RNP complexes in Gi-NS-PR8-infected cells compared to Gi-wt-infected cells. Nevertheless, a similar RNP export kinetic was observed (Supplementary Figure S2).

### Potential of Different NS1 Proteins to Regulate the IFN-β Promoter

**FIGURE 3:**
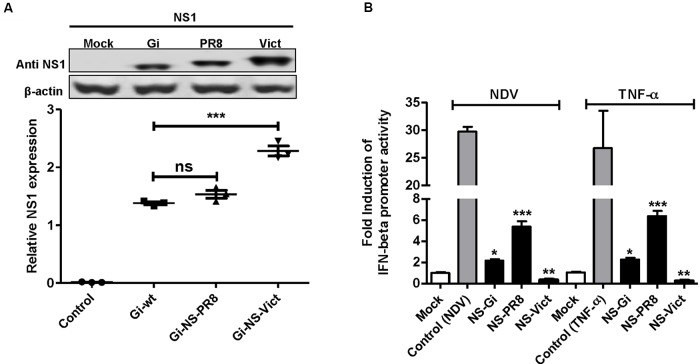
Viral expression of different NS1 proteins and their ability to regulate IFN-β induction. **(A)** NS1 expression 24 h p.i. in A549 infected with Gi-wt, Gi-NS-PR8, and Gi-NS-Vict (MOI of 1) was detected by Western blot. **(B)** Effect on IFN-β promoter-induction by transiently expressed NS1 of Gi-wt (NS-Gi), PR8 (NS-PR8) and Victoria (NS-Vict) was analyzed. 293T cells were transfected with pMP-NS-Gi, pMP-NS-PR8, pMP-NS-Vict or empty pHW2000 (Mock and control) together with a Firefly luciferase expression plasmid under the control of IFN-β promoter (p125-Luc) and *Renilla* luciferase expression plasmid (pRL-SV40). Except for the mock, the transfected cells were either infected with NDV (MOI = 3) or treated with 50 ng/ml recombinant human TNF-α for the induction of IFN-β promoter. After 24 h p.i. the cells were lysed and assayed for luciferase activity. Statistical analysis was performed using “student *t*-test” test. ^∗^*p* < 0.05, ^∗∗^*p* < 0.001, ^∗∗∗^*p* < 0.0001; ns, non-significant. Error bars represent standard deviation (SD).

The ability of NS1 protein to antagonize the virus-induced antiviral cell response supports replication of IAV (Hale et al., 2008). We therefore measured the relative amount of NS1 protein expressed by the recombinant Gi-wt, Gi-NS-PR8, and Gi-NS-Vict in A549 cells. Western blotting analysis showed that NS1 proteins were all expressed and displayed as a single band of the expected molecular weight (25–26 kDa). Yet, we observed a significantly stronger accumulation of NS1 of Gi-NS-Vict than of NS1 protein from Gi-wt and Gi-NS-PR8 (**Figure 3A**). Nevertheless, it should be noted that the signal strength for the different NS1 proteins expressed could also originate from differential recognition by the monoclonal antibody that was used.

Next, the ability of different NS1 proteins to control the induction of the IFN-β promoter was tested. To this point, plasmids encoding the NS segments of Gi-wt (NS-Gi), PR8 (NS-PR8), and Victoria (NS-Vict) were each co-transfected into human 293T cells together with a plasmid expressing luciferase under control of the IFN-β promoter. Cells transfected with empty vector (no NS1 gene) were either used as a mock (non-stimulated) or as a control (stimulated). All transfected cells except “mock” were followed by induction of the IFN-β promoter, either with Newcastle disease virus-infection (MOI = 3) (Hayman et al., 2006; Shelton et al., 2012) or treatment with recombinant human TNF-α (50 ng/ml) (Osterlund et al., 2005). Unlike NS1-Gi, NS1-PR8 or NS1-Vict expressing cells, NDV infection or TNF-α treatment of the control induced robust IFN-β promoter-driven Firefly luciferase activity. This is consistent with the commonly accepted IFN-antagonistic activity of NS1. Nevertheless, compared to the non-stimulated mock-transfected cells, a two or fivefold IFN-β promoter induction was still observed in stimulated NS1-Gi or NS1-PR8-expressing cells, respectively, while NS1-Vict expression prevented IFN-β induction (**Figure 3B**). These data indicate that in this setting NS1-Gi has a better capability to antagonize IFN-β induction than NS1-PR8, whereas NS1-Vict seems to suppress IFN-β induction completely, when compared to the mock. Therefore, the increased replication of Gi-NS-PR8 is not connected to a fully reduced IFN induction.

### Cytokine, Chemokine, and IFN-Related mRNA Expression Profiles in A549 and dTHP-1 Cells

It was previously shown that cytokine-, chemokine-, and IFN-related gene products induced by IAV infection control viral propagation (Mukherjee et al., 2011; Ishikawa, 2012) and that specific IAVs (pandemic H1N1_1918_ and H5N1-type highly pathogenic avian influenza virus) induce a strong over expression of such factors, causing a so called cytokine storm (CS). CS can lead to increased pathogenicity and severe disease in infected humans (Ishikawa, 2012; Tisoncik et al., 2012; Ranaware et al., 2016). This was observed for infected airway epithelial cells supporting productive viral replication, as well as alveolar macrophages and dendritic cells, which do not support productive IAV infections (Perrone et al., 2008; Gill et al., 2010). Similar to airway epithelial cells, human macrophages express type I and type III IFNs, IL-1α, IL-1β, IL-6, TNF-α, CXCL8, CCL2 (MCP-1), CCL3 (MIP-1α), CCL4 (MIP1-β), CXCL9, and CXCL10 following infection with seasonal H1N1 or with H5N1, 2009 H1N1 strains demonstrating increased pathogenicity (Geiler et al., 2011).

**FIGURE 4:**
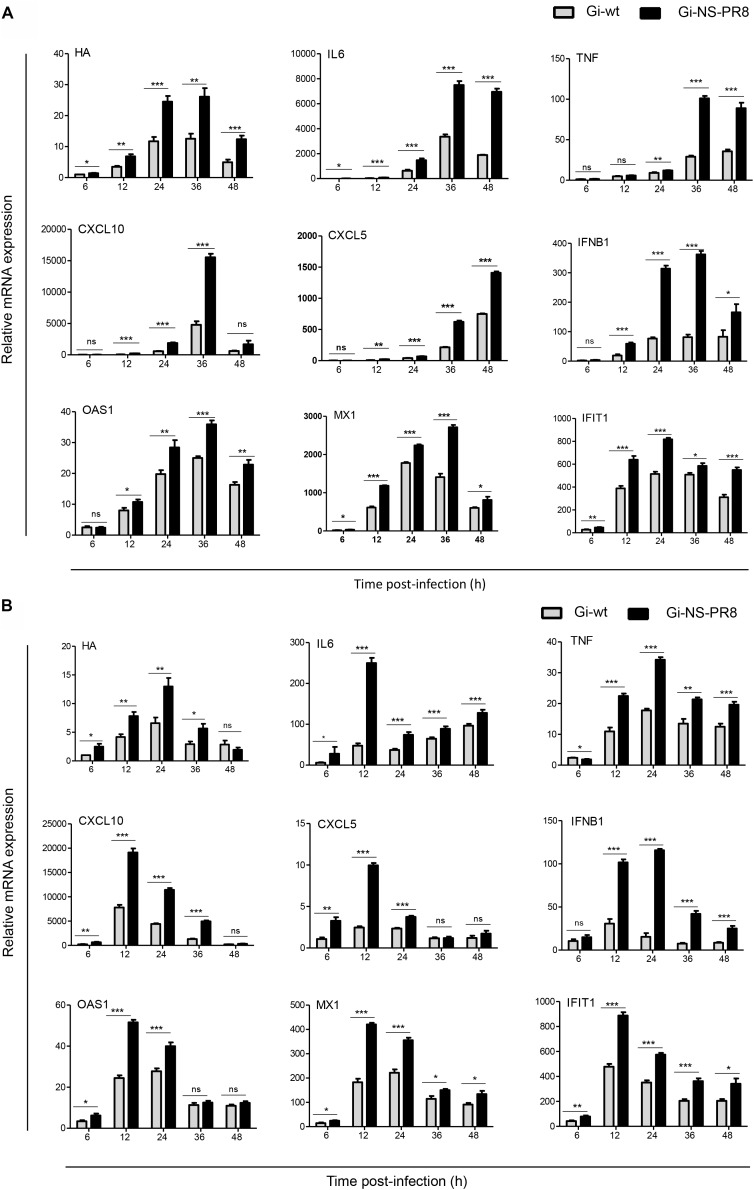
Differential induction of host cell cyto-/chemokines and IFN-related genes during infection of human cell lines with Gi-wt or Gi-NS-PR8 viruses. **(A)** A549 cells. **(B)** dTHP-1 cells. mRNA expression was determined by RT-qPCR and normalized to β-actin mRNA. Data were pooled from two independent experiments with three replicates (final *n* = 6). HA mRNA levels are shown relative to expression by Gi-wt at 6 h p.i., which was arbitrarily assigned a value of 1. Expression of the cellular mRNAs is shown relative to expression in uninfected cells (*t* = 0 h), which was assigned a value of 1. Statistical analysis was performed using “two-way ANOVA,” followed by “Bonferroni *post hoc*” test. ^∗^*p* < 0.05, ^∗∗^*p* < 0.001, ^∗∗∗^*p* < 0.0001; ns, non-significant. Significance of differences at individual time points was assessed with student *t*-test: ^∗^*p* < 0.05, ^∗∗^*p* < 0.001, ^∗∗∗^*p* < 0.0001. Error bars represent standard error mean (±SEM).

As NS1 is the main viral factor controlling cellular innate immune response in order to promote viral replication (Mukherjee et al., 2011; Ishikawa, 2012) we next examined (i) whether the growth advantage in A549 (human lung epithelia) cells conferred by the NS segment of PR8 might be associated with altered mRNA expression levels of antiviral acting cyto-/chemokines- and interferon-related cellular genes, and (ii) whether NS segment-specific effects would also be evident in dTHP-1 cells. These cells are human monocytic THP-1 cells, which were morphologically and functionally differentiated to non-productive macrophages (Short et al., 2012). **Figure 4A** shows results obtained from infected A549 cells. Consistent with the increased viral titres observed for Gi-NS-PR8 in this cell line (**Figure 1**) viral transcription, as measured by IAV hemagglutinin (HA) mRNA expression level, was also markedly higher in Gi-NS-PR8-infected cells as early as 12 h p.i.. Interestingly, Gi-NS-PR8 infection also resulted in significantly higher induction of all cytokines (IL-6 and TNF-β), chemokines (CXCL10 and CXCL5), and IFN-related genes (IFN-β, OAS, MX1, and IFIT1) assayed.

Results of dTHP-1 cell infection are shown in **Figure 4B**. Compared to Gi-wt, a significant higher transcription of Gi-NS-PR8 HA mRNA was detected as early as 6 h p.i., which increased to ∼2-fold higher expression levels by 24 h p.i.. HA transcription level of both strains subsided thereafter, which is consistent with a non-productive infection. Transcription of the host cell targets followed similar kinetics, as highest levels were detected at 12 or 24 h p.i.. Again, induction of all host mRNA targets assayed was significantly higher in Gi-NS-PR8 infected cells.

Thus, reassortment of the PR8 NS segment resulted in an increased transcription of viral and host cell mRNAs in both productive and non-productive infection, indicating a lower potential of the PR8 NS segment to control cellular innate immune responses compared to the Gi-wt NS segment. Nevertheless, the replication efficiency of Gi-NS-PR8 does not seem to be dampened by expression of antiviral acting cellular factors.

### Virulence of Recombinant Gi-wt and Gi-NS-PR8 in Mice

**FIGURE 5:**
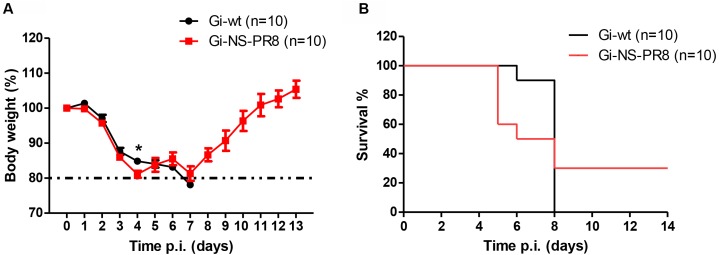
Analysis of viral pathogenesis *in vivo*. Groups of 10 female C57BL/6J mice (10–12 weeks of age) were infected intra-nasally with 5 × 10^5^ FFU of Gi-wt or Gi-NS-PR8 virus. **(A)** Calculated percentages of body weight loss was recorded up to 14 days p.i.. Differences in weight loss between infection with Gi-wt and with Gi-NS-PR8 were significant (*p* < 0.025, repeated measures ANOVA) for days 1–4. Error bars indicate means ± SEM. **(B)** Percentages of mice surviving at the indicated time points (up to 14 days p.i.). Mice had to be euthanized when weight loss exceeded 20%. Asterisk (^∗^) refers to significant difference of means.

To investigate whether the PR8 NS would affect virulence in a mammalian host, C57BL/6J mice (*n* = 10 per group) were infected with either Gi-wt or Gi-NS-PR8 (5 × 10^5^ FFU/animal) (**Figure 5**). At day 4 p.i., we observed a strong weight loss among the Gi-NS-PR8-infected mice (**Figure 5A**) necessitating euthanasia of four mice (**Figure 5B**). Three additional Gi-NS-PR8-infected mice had to be euthanized by day 7 due to excessive weight loss. The remaining three Gi-NS-PR8-infected mice recovered and regained their body weight between days 10 and 12. In contrast, all mice infected with Gi-wt continued to lose weight after day 4 and weight loss exceeded 20% of all 10 mice by day 7 (**Figure 5A**), necessitating euthanasia (**Figure 5B**). Therefore, Gi-NS-PR8 exhibited similar virulence as Gi-wt during the early stages of infection, but as 30% of the Gi-NS-PR8-infected mice recovered, the increased replication efficiency of Gi-NS-PR8 *in vitro* did not result in an increased mortality *in vivo* in this mouse model.

### Replication of Recombinant Gi-wt and Gi-NS-PR8 in Avian Cells

**FIGURE 6:**
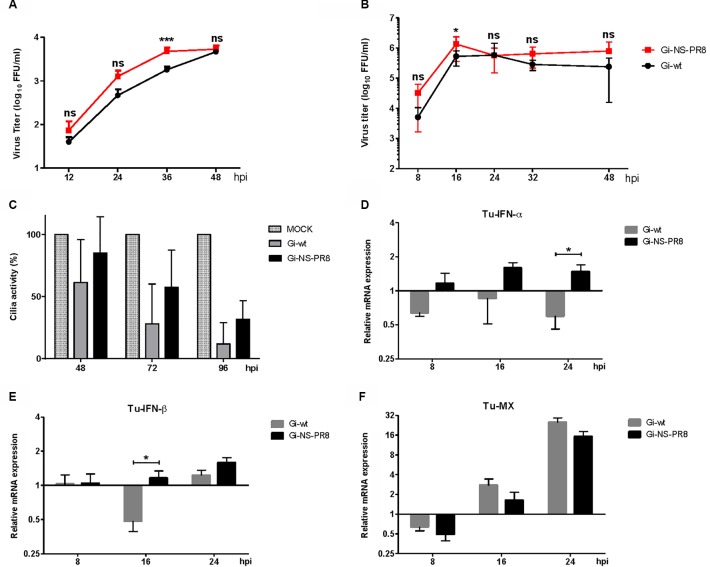
Analysis of Gi-wt- and Gi-NS-PR8-infected QT6 cells and avian tracheal organ cultures. Tracheal organ cultures (TOC) of turkey were each inoculated with 10^3^ FFU of viruses (*n* = 6/group). **(A)** Replication kinetics for Gi-wt and Gi-NS-PR8 viruses in QT6 cells, infected at an MOI of 0.001, were done at 12, 24, 36, and 48 h p.i.. Viral titers for the indicated time points were harvested and assayed by foci assay (FFU/ml). The titers were calculated for three independent experiments. Error bars represent standard deviation (SD). **(B)** Viral titers of the TOC supernatants are presented in growth curves; error bars represent means ± SD. **(C)** Ciliostasis of TOC’s ciliated epithelium is shown in mean percent with SD. **(D–F)** Comparative levels of TuINFα, TuIFNβ, and TuMX mRNA expression normalized to 28S rRNA expression. Error bars represent means ± SEM. Asterisk (^∗^) refers to significant difference of means. Statistical analysis was performed using “Student *t*-test” and “repeated measures ANOVA,” followed by “Bonferroni *post hoc*” test. ^∗^*p* < 0.05, ^∗∗^*p* < 0.001, ^∗∗∗^*p* < 0.0001; ns, non-significant.

Poultry species, except quails and turkey, show limited ability to replicate and transmit the H1N1pdm09 (Ilyushina et al., 2010; Pantin-Jackwood et al., 2010). Quails are broadly susceptible to natural infection with several mammalian and avian IAV subtypes (Ilyushina et al., 2010). Therefore, we chose the quail QT6 cells to investigate the impact of the NS-PR8 on Gi-wt and found that the replication efficiency of Gi-NS-PR8 was significantly increased when compared to Gi-wt (**Figure 6A**).

Since replication of Gi-NS-PR8 was increased in quail QT6 cells *in vitro*, viral growth kinetics were furthermore tested *ex vivo* in tracheal organ cultures (TOCs) of turkeys (TOC-Tu), which are known to be more susceptible to H1N1pdm09 compared to chickens (Pantin-Jackwood et al., 2010). Successful replication of both Gi-wt and Gi-NS-PR8 was observed in TOC-Tu derived from the upper respiratory tract airway epithelium.

Interestingly, replication efficiency was slightly higher in Gi-NS-PR8-infected TOC-Tu compared to Gi-wt infection at different time points analyzed (except for 24 h p.i.), which was significant at 16 h pi (*P* < 0.05) (**Figure 6B**). However, development of ciliostasis of the TOC epithelium as a result of necrosis and apoptosis did not differ significantly between the two groups (**Figure 6C**). Cilia activity started to decrease at 48 h p.i. in both Gi-wt- and Gi-NS-PR8-infected groups, and dropped further to less than 30% at 96 h p.i..

In accordance with data obtained from infected mammalian cells (A549 and dTHP-1), mRNA transcription levels of turkey IFN-α (TuIFN-α; **Figure 6D**) and turkey IFN-β (TuIFN-β; **Figure 6E**) were increased in Gi-NS-PR8-infected TOC-Tu compared to non-infected controls at 8, 16, and 24 h p.i.. Interestingly, mRNA amount of TuIFN-α was significantly decreased in Gi-wt-infected TOC-Tu at 8 and 24 h p.i., which was also observed for TuIFN-β at 16 h p.i. (*P* < 0.05). However, we observed no significant difference in the mRNA expression level of interferon-stimulated turkey MX gene (TuMX) between the two virus-infected TOC-Tu groups (**Figure 6F**). After initial suppression at 8 h p.i., both Gi-wt and Gi-NS-PR8 showed similar increases in TuMX mRNA transcription rate at 16 and 24 h p.i., compared to virus-free TOC. These results demonstrate that the effect of the PR8 NS on the virus-induced expression of antiviral factors is not restricted to mammals and that similar to the enhanced propagation of Gi-NS-PR8 in human cell culture the replication efficiency was enhanced in susceptible turkey TOC primary cultures despite significantly up-regulated mRNA transcripts for interferons (α/β).

### Virulence of Recombinant Gi-wt and Gi-NS-PR8 in Chickens

It was previously demonstrated that chickens were refractory to infection with H1N1pdm09 (Kalthoff et al., 2010). To analyze whether the PR8 NS segment would increase the virulence of Gi-NS-PR8, 6-week-old SPF chickens were inoculated with both recombinant reassortant viruses, respectively. None of the chickens in either group showed any clinical signs within 21 days post inoculation with 10^6^ FFU per bird via the oculo-oronasal route. All oropharyngeal and cloacal swab samples of inoculated as well as sentinel chickens were negative for virus RNA by H1-specific RT-qPCR at 1–7 days p.i.. None of the birds seroconverted by 21 days p.i., as tested with an IAV NP-specific ELISA (data not shown). These results indicate that the PR8 NS did not confer improved replication ability to Gi-NS-PR8 in chicken.

## Discussion

The expanding geographic distribution and continuous evolution of H1N1pdm09 and its circulation in swine and human populations raises concerns about possible reassortment with other IAVs that might result in variants with increased pathogenicity and/or a change of host range (Ma et al., 2015; Nelson et al., 2015). Although the descendants of the pandemic H1N1_1918_ virus (including A/PR/8/1934, H1N1) appear to be completely replaced by the pandemic 2009 H1N1 strain, phylogenetic and geographic analyses revealed the global prevalence of avian IAV genes whose proteins differ only by a few amino acids (aa) from the H1N1_1918_ strain, suggesting that 1918-like pandemic viruses may emerge in the future (Watanabe et al., 2014). This might be fostered by the reassortment between H1N1pdm09 and other (avian) IAVs carrying PR8-like NS segments. The PR8 NS1 and NEP/NS2 proteins differ from those of H1N1_1918_ at 12 (3P, 22V, 81M, 100E, 103S, 106I, 114P, 171A, 215T, 221E, 224G, and 227R) and 9 (3P, 14L, 22E, 63E, 70G, 86K, 89V, and 104H) aa positions, respectively. These aa differences between the H1N1_1918_, and PR8 NS1 were previously shown to be important in the adaptation and virulence of PR8 in mice compared to the H1N1_1918_ strain (Basler et al., 2001). To this point we had included PR8 NS as a representative of an H1N1_1918_-like segment with an adaptive and virulent trait in mice.

The NS1 protein was previously reported to play an important role for IAV replication and host range through different interactions with cellular proteins (Hale et al., 2008; Ma et al., 2010; Wang et al., 2010; Kanrai et al., 2016). Previous studies have shown that single NS-reassortment can confer significant changes to (i) the viral growth kinetic, (ii) the host innate immune response and (iii) pathogenicity in mammals without prior adaptation (Ma et al., 2010; Wang et al., 2010; Petersen et al., 2013; Kanrai et al., 2016). Our *in vitro* and *in vivo* investigation of the characteristics of the Gi-reassortants containing the NS segments from several different IAV subtypes in comparison with Gi-wt, demonstrated that only the PR8 NS segment in the GI-NS-PR8 could concurrently provoke higher replication efficiency and elevated levels of interferon-stimulated gene expression in mammalian and avian cells.

Phylogenetic analysis revealed that NS1 of PR8 displays a homology to NS1 proteins of human H1- and H3-type IAV as well as to those of avian H5-, H7- and H9-type IAV (Supplementary Figure S3). Therefore, it might possess features found in mammalian and avian IAV type NS1 proteins. In this respect, it is important to note that the PR8 NS segment enhances viral replication efficiency in mammalian A549 and NPTr cells when introduced into the genetic backbone of the pandemic 2009 isolate Gi-wt and also provides increased replication ability of GI-NS-PR8 in susceptible avian cells (QT6, TOC-Tu).

Although apoptosis is one of the host defense strategies to limit replication of invading IAVs, several studies have suggested that IAV might induce apoptotic signaling pathways for the benefit of viral replication, spread and pathogenicity (Herold et al., 2012; Wang et al., 2014; Muhlbauer et al., 2015). Yet, the increased replication efficiency of Gi-NS-PR8 (**Figure 1**) was not found to be correlated with an altered apoptotic activity when compared to Gi-wt-infected cells at late stages of the viral replication cycle (10 h p.i., **Figure 2**). Also, variations between the NEP/NS2 of PR8 and Gi-wt did not seem to impact on the nuclear vRNP export kinetics (Supplementary Figure S2). As these results argue against a differential control of virus-induced apoptosis and nuclear RNP export by the PR8 NS segment, the observed differential replication efficiencies between Gi-wt and Gi-NS-PR8 have to be due to other reasons.

Suppression of IFN induction is one of the mechanisms that IAV employ to enhance their replication (Marcus et al., 2005), and our data demonstrate that both transiently expressed NS1 of Gi-wt and PR8-wt cannot efficiently control the induction of the IFN-β promoter by NDV infection or TNF-α treatment. Nevertheless, a statistically significant difference in the IFN-β promoter induction between NS1-Gi and NS1-PR8 protein expressing cells is observed (**Figure 3B**). Interestingly, the obtained data were comparable for IFN-β promoter activation with the NDV infection and for TNF-α treatment.

The observation that a specific IAV allows high IFN-β expression, but at the same time does not show impaired replication efficiency was previously reported in several other studies *in vitro* and *in vivo*. The authors referred this phenomenon mainly to unique residues in NS1, which affect its binding ability to important cellular interactors like CPSF-30 (Dankar et al., 2011, 2013; Meunier et al., 2012; Shelton et al., 2012). The CPSF-30 is involved in the maturation of host cell mRNAs. To hijack the CPSF-30 function the NS1 interacts with the cellular CPSF-30 mainly via the effector domain (ED; residues 88–202) of NS1 and two zinc fingers domains of CPSF-30 (the F2F3 region) (Twu et al., 2006; Das et al., 2008; Ai et al., 2014). NS1 and CPSF-30 form a complex with the viral polymerase subunits to allow 3′-end processing of viral mRNAs (Kuo and Krug, 2009). It was suggested that NS1/CPSF-30 interaction might affect polymerase activity limiting viral RNA accumulation. Therefore, viruses with reduced NS1-binding to CPSF-30 would gain an increased polymerase activity, resulting in higher vRNA yields and allowing them to replicate despite a moderate cytokine response. (Shelton et al., 2012). It was previously reported that a mouse-adapted variant of the pandemic virus A/Hong Kong/68 had acquired two mutations, F103L and M106I, which led to impaired interaction of NS1 with CPSF-30 (Dankar et al., 2011). This mouse-adapted H3N2 virus showed an increased replication capacity despite the fact that it also induced high levels of IFN. Similarly, comparing the aa-sequences of NS1-Gi and -PR8, we found that NS1-PR8 possesses a serine at aa position 103 (phenylalanine in NS1-Gi) and isoleucine at position 106 (methionine at NS1-Gi) (data not shown), which might be a reason for the impaired binding capacity of NS1-PR8 to CPSF-30 that has been previously observed (Hale et al., 2008). Despite the fact that NS1-Gi possesses the important aa residues 103F and 106M to interact with CPSF-30, it also accommodates three aa residues (108R, 125E, and 189G), which were shown to impair the interaction of CPSF-30 with NS1 protein from H1N1pdm09 A/California/04/2009 (H1N1, NS1-Cal/09) (Hale et al., 2010). This might explain why NS1-Gi cannot fully suppress IFN-induction. Regarding the stronger suppression of the IFN-β promoter by NS1-Gi compared to NS1-PR8, it should be noted that NS1-Gi and NS1-Cal/09 differ only in two aa positions, 123: V/I and 133: D/N, respectively. Interestingly, the 123V (not present in the NS1-PR8) was recently shown to partially restore the ability of NS1-Cal/9 to interact with the CPSF-30 and to certain extend limit general host gene expression (Clark et al., 2017). Interestingly, the currently circulating descendants of H1N1pdm09 have gradually gained six aa changes (55K, 90I, 123V, 125D, 131E, and 205S) in their NS1 protein. These aa residues promote the full restoration to efficiently bind to CPSF-30, and thereby actively inhibit host gene expression (Clark et al., 2017). Notably, the NS1-Vict possesses: 103F, 106M, 108K, 123I, 125D, and 189D. Taken together, these aa differences could provide a possible explanation for the differential impact of NS1-Gi and NS1-PR8 on IFN-β promoter induction.

*In vivo*, the Gi-NS-PR8 virus exhibited similar virulence in mice by the 4th day p.i. compared to the Gi-wt. The onset of mortality was earlier yet, in contrast to Gi-wt-infected mice, 30% of Gi-NS-PR8-infected mice survived. At the moment we can only speculate that an increased cytokine induction, as observed for the infected A549 cells, might have promoted pathogenesis and disease development at the early stage of infection (days 1–4) as seen for highly pathogenic and pandemic IAV strains (Kobasa et al., 2004; Tumpey et al., 2005; de Jong et al., 2006; Loo and Gale, 2007; Szretter et al., 2007), but could also have assisted to the survival of the mice at later stages (days 8–14). In a recent study, Turnbull et al. (2016) compared pathogenicity and induction of host innate immune response following mice infection with PR8-wt and PR8-reassortants in which the PR8 NS segment was replaced with allele A and allele B NS segments, derived from a variety of North American LPAI virus strains collected between 1986 and 2005. Interestingly, compared to other PR8-NS-reassortants, the authors demonstrated that the PR8-wt could promote superior replication efficiency in mammalian cells, increased pathogenicity and elevated levels of interferon-stimulated genes expression in mice (Turnbull et al., 2016).

Chickens are known to be resistant to wild-type H1N1pdm09 (Swayne et al., 2009; Kalthoff et al., 2010). In our present study, susceptibility of chickens to the recombinant isolate Gi-wt was not increased by NS-reassortment of PR8. However, recent *in vivo* experiments showed that single replacement of specific segments (including the NS segment) of a pandemic H1N1 strain (A/Beijing/16/2009) by viral segments of A/chicken/Hebei/LC/2008 (H9N2) enabled the reassortant viruses to transmit/replicate in chickens, which are otherwise refractory to infection with the wild-type virus (Sun et al., 2015). Taken together, these results implicate that the ability of H1N1pdm09 to cross the species barrier to chickens after NS-reassortment is based on the specific genetic backbone of the respective H1N1pdm09 strain, as well as the particular NS segment/-donor strain.

Unlike chicken, turkeys are susceptible to a wide variety of IAVs including swine and human-adapted strains, such as H1N1pdm09 (Choi et al., 2004; Mathieu et al., 2010). H1N1pdm09 outbreaks in turkey flocks were frequently reported in several countries (Swayne et al., 2009). This makes turkeys a potential “mixing vessel” for H1N1pdm09. TOC represent an excellent *ex vivo* system allowing to study infection of the upper respiratory tract of birds (Zaffuto et al., 2008). The increase of type-I IFN mRNA expression in Gi-NS-PR8-infected TOC-Tu mirrors the findings of the experiments in mammalian cell cultures. However, no increase in virulence as measured by ciliostasis was observed (Zaffuto et al., 2008).

## Conclusion

The introduction of the NS-segment from PR8 (H1N1) into the genetic backbone of a 2009 pandemic H1N1 IAV strain (A/Giessen/06/09) was shown to increase replication efficiency of the reassortant virus in human, porcine and avian respiratory cells. The increased expression of chemokines, cytokines and ISGs may eventually also enhance virulence in mammalian hosts. The results indicate a strong potential of H1N1pdm09 to increase its replication ability in mammalian and in susceptible avian systems. Even though pathogenicity in chicken, mice and turkey TOC was not augmented, the improved replication ability in mammalian cell lines and turkey TOC could provide the possibility for faster adaptation, which might eventually lead to stronger pathogenicity. Therefore, close observation of the genetic development of H1N1pdm09 strains is warranted.

## Author Contributions

HP, AM, MB, SR, and SP conceived and designed the experiments. HP, AM, MT, AI, DH, AT, and BS performed the experiments. HP, AM, MT, AI, DH, AT, FP, MB, SR, and SP analyzed the data. FP, MB, SR, and SP contributed reagents/materials/analysis tools. HP, AM, FP, SR, and SP wrote the paper. HP, AM, DH, FP, MB, SR, and SP revised the final manuscript. All authors reviewed the manuscript.

## Conflict of Interest Statement

The authors declare that the research was conducted in the absence of any commercial or financial relationships that could be construed as a potential conflict of interest. The handling Editor declared a shared affiliation, though no other collaboration, with several of the authors DH and MB.

## References

[B1] Abdelwhab E. M. Hassan M. K. Abdel-Moneim A. S. Naguib M. M. Mostafa A. Hussein I. T. (2016). Introduction and enzootic of A/H5N1 in Egypt: virus evolution, pathogenicity and vaccine efficacy ten years on. *Infect. Genet. Evol.* 40 80–90. 10.1016/j.meegid.2016.02.023 26917362

[B2] Ai H. Zhang L. Chang A. K. Wei H. Che Y. Liu H. (2014). Virtual screening of potential inhibitors from TCM for the CPSCPSF30 binding site on the NS1A protein of influenza A virus. *J. Mol. Model.* 20:2142. 10.1007/s00894-014-2142-7 24562912

[B3] Basler C. F. Reid A. H. Dybing J. K. Janczewski T. A. Fanning T. G. Zheng H. (2001). Sequence of the 1918 pandemic influenza virus nonstructural gene (NS) segment and characterization of recombinant viruses bearing the 1918 NS genes. *Proc. Natl. Acad. Sci. U.S.A.* 98 2746–2751. 10.1073/pnas.031575198 11226311 PMC30210

[B4] Bibert S. Roger T. Calandra T. Bochud M. Cerny A. Semmo N. (2013). IL28B expression depends on a novel TT/-G polymorphism which improves HCV clearance prediction. *J. Exp. Med.* 210 1109–1116. 10.1084/jem.20130012 23712427 PMC3674704

[B5] Chakrabarti A. K. Vipat V. C. Mukherjee S. Singh R. Pawar S. D. Mishra A. C. (2010). Host gene expression profiling in influenza A virus-infected lung epithelial (A549) cells: a comparative analysis between highly pathogenic and modified H5N1 viruses. *Virol. J.* 7:219. 10.1186/1743-422X-7-219 20828378 PMC2945955

[B6] Choi Y. K. Lee J. H. Erickson G. Goyal S. M. Joo H. S. Webster R. G. (2004). H3N2 influenza virus transmission from swine to turkeys, United States. *Emerg. Infect. Dis.* 10 2156–2160. 10.3201/eid1012.040581 15663853 PMC3323362

[B7] Clark A. M. Nogales A. Martinez-Sobrido L. Topham D. J. Dediego M. L. (2017). Functional evolution of influenza virus NS1 protein in currently circulating human 2009 pandemic H1N1 viruses. *J. Virol.* 91:e00721-17. 10.1128/JVI.00721-17 28637754 PMC5553169

[B8] Dankar S. K. Miranda E. Forbes N. E. Pelchat M. Tavassoli A. Selman M. (2013). Influenza A/Hong Kong/156/1997(H5N1) virus NS1 gene mutations F103L and M106I both increase IFN antagonism, virulence and cytoplasmic localization but differ in binding to RIG-I and CPSCPSF30. *Virol. J.* 10:243. 10.1186/1743-422X-10-243 23886034 PMC3733596

[B9] Dankar S. K. Wang S. Ping J. Forbes N. E. Keleta L. Li Y. (2011). Influenza A virus NS1 gene mutations F103L and M106I increase replication and virulence. *Virol. J.* 8:13. 10.1186/1743-422X-8-13 21226922 PMC3032709

[B10] Das K. Ma L. C. Xiao R. Radvansky B. Aramini J. Zhao L. (2008). Structural basis for suppression of a host antiviral response by influenza A virus. *Proc. Natl. Acad. Sci. U.S.A.* 105 13093–13098. 10.1073/pnas.0805213105 18725644 PMC2522260

[B11] de Jong M. D. Simmons C. P. Thanh T. T. Hien V. M. Smith G. J. Chau T. N. (2006). Fatal outcome of human influenza A (H5N1) is associated with high viral load and hypercytokinemia. *Nat. Med.* 12 1203–1207. 10.1038/nm1477 16964257 PMC4333202

[B12] Fan S. F. Macken C. A. Li C. J. Ozawa M. Goto H. Iswahyudi N. F. N. (2013). Synergistic effect of the PDZ and p85 beta-binding domains of the NS1 protein on virulence of an avian H5N1 influenza A virus. *J. Virol.* 87 4861–4871. 10.1128/JVI.02608-12 23408626 PMC3624326

[B13] Gannage M. Dormann D. Albrecht R. Dengjel J. Torossi T. Ramer P. C. (2009). Matrix protein 2 of influenza A virus blocks autophagosome fusion with lysosomes. *Cell Host Microbe* 6 367–380. 10.1016/j.chom.2009.09.005 19837376 PMC2774833

[B14] Garaigorta U. Falcon A. M. Ortin J. (2005). Genetic analysis of influenza virus NS1 gene: a temperature-sensitive mutant shows defective formation of virus particles. *J. Virol.* 79 15246–15257. 10.1128/JVI.79.24.15246-15257.2005 16306596 PMC1316024

[B15] Geiler J. Michaelis M. Sithisarn P. Cinatl J. Jr. (2011). Comparison of pro-inflammatory cytokine expression and cellular signal transduction in human macrophages infected with different influenza A viruses. *Med. Microbiol. Immunol.* 200 53–60. 10.1007/s00430-010-0173-y 20865277

[B16] Gill J. R. Sheng Z. M. Ely S. F. Guinee D. G. Beasley M. B. Suh J. (2010). Pulmonary pathologic findings of fatal 2009 pandemic influenza A/H1N1 viral infections. *Arch. Pathol. Lab. Med.* 134 235–243. 10.1043/1543-2165-134.2.235 20121613 PMC2819217

[B17] Hale B. G. (2014). Conformational plasticity of the influenza A virus NS1 protein. *J. Gen. Virol.* 95 2099–2105. 10.1099/vir.0.066282-0 24928909

[B18] Hale B. G. Randall R. E. Ortin J. Jackson D. (2008). The multifunctional NS1 protein of influenza A viruses. *J. Gen. Virol.* 89 2359–2376. 10.1099/vir.0.2008/004606-0 18796704

[B19] Hale B. G. Steel J. Medina R. A. Manicassamy B. Ye J. Hickman D. (2010). Inefficient control of host gene expression by the 2009 pandemic H1N1 influenza A virus NS1 protein. *J. Virol.* 84 6909–6922. 10.1128/JVI.00081-10 20444891 PMC2898253

[B20] Hayman A. Comely S. Lackenby A. Murphy S. Mccauley J. Goodbourn S. (2006). Variation in the ability of human influenza A viruses to induce and inhibit the IFN-beta pathway. *Virology* 347 52–64. 10.1016/j.virol.2005.11.024 16378631

[B21] Herold S. Ludwig S. Pleschka S. Wolff T. (2012). Apoptosis signaling in influenza virus propagation, innate host defense, and lung injury. *J. Leukoc. Biol.* 92 75–82. 10.1189/jlb.1011530 22345705

[B22] Hoffmann B. Harder T. Lange E. Kalthoff D. Reimann I. Grund C. (2010). New real-time reverse transcriptase polymerase chain reactions facilitate detection and differentiation of novel A/H1N1 influenza virus in porcine and human samples. *Berl. Munch. Tierarztl. Wochenschr.* 123 286–292. 20690540 10.2376/0005-9366-123-286

[B23] Hoffmann E. Neumann G. Kawaoka Y. Hobom G. Webster R. G. (2000). A DNA transfection system for generation of influenza A virus from eight plasmids. *Proc. Natl. Acad. Sci. U.S.A.* 97 6108–6113. 10.1073/pnas.100133697 10801978 PMC18566

[B24] Hoffmann E. Stech J. Guan Y. Webster R. G. Perez D. R. (2001). Universal primer set for the full-length amplification of all influenza A viruses. *Arch. Virol.* 146 2275–2289. 10.1007/s007050170002 11811679

[B25] Hutchinson E. C. Charles P. D. Hester S. S. Thomas B. Trudgian D. Martinez-Alonso M. (2014). Conserved and host-specific features of influenza virion architecture. *Nat. Commun.* 5:4816. 10.1038/ncomms5816 25226414 PMC4167602

[B26] Ilyushina N. A. Kim J. K. Negovetich N. J. Choi Y. K. Lang V. Bovin N. V. (2010). Extensive mammalian ancestry of pandemic (H1N1) 2009 virus. *Emerg. Infect. Dis.* 16 314–317. 10.3201/eid1602.091141 20113569 PMC2958019

[B27] Ishikawa T. (2012). Clinical preparedness for cytokine storm induced by the highly pathogenic H5N1 influenza virus. *J. Pharmacogenomics Pharmacoproteomics* 3:e131 10.4172/2153-0645.1000e131

[B28] Jin L. Lenz L. L. Cambier J. C. (2010). Cellular reactive oxygen species inhibit MPYS induction of IFNbeta. *PLoS One* 5:e15142. 10.1371/journal.pone.0015142 21170271 PMC3000824

[B29] Kaiser P. Rothwell L. Galyov E. E. Barrow P. A. Burnside J. Wigley P. (2000). Differential cytokine expression in avian cells in response to invasion by *Salmonella typhimurium*, *Salmonella enteritidis* and *Salmonella gallinarum*. *Microbiology* 146(Pt 12), 3217–3226. 10.1099/00221287-146-12-3217 11101679

[B30] Kalthoff D. Grund C. Harder T. C. Lange E. Vahlenkamp T. W. Mettenleiter T. C. (2010). Limited susceptibility of chickens, Turkeys, and Mice to Pandemic (H1N1) 2009 Virus. *Emerg. Infect. Dis.* 16 703–705. 10.3201/eid1604.091491 20350393 PMC3321957

[B31] Kanrai P. Mostafa A. Madhugiri R. Lechner M. Wilk E. Schughart K. (2016). Identification of specific residues in avian influenza A virus NS1 that enhance viral replication and pathogenicity in mammalian systems. *J. Gen. Virol.* 97 2135–2148. 10.1099/jgv.0.000542 27405649

[B32] Kapadia S. B. Brideau-Andersen A. Chisari F. V. (2003). Interference of hepatitis C virus RNA replication by short interfering RNAs. *Proc. Natl. Acad. Sci. U.S.A.* 100 2014–2018. 10.1073/pnas.252783999 12566571 PMC149950

[B33] Kobasa D. Takada A. Shinya K. Hatta M. Halfmann P. Theriault S. (2004). Enhanced virulence of influenza A viruses with the haemagglutinin of the 1918 pandemic virus. *Nature* 431 703–707. 10.1038/nature02951 15470432

[B34] Krumbholz A. Philipps A. Oehring H. Schwarzer K. Eitner A. Wutzler P. (2011). Current knowledge on PB1-F2 of influenza A viruses. *Med. Microbiol. Immunol.* 200 69–75. 10.1007/s00430-010-0176-8 20953627

[B35] Kuo R. L. Krug R. M. (2009). Influenza A virus polymerase is an integral component of the CPSCPSF30-NS1A protein complex in infected cells. *J. Virol.* 83 1611–1616. 10.1128/JVI.01491-08 19052083 PMC2643760

[B36] Lai S. J. Qin Y. Cowling B. J. Ren X. Wardrop N. A. Gilbert M. (2016). Global epidemiology of avian influenza A H5N1 virus infection in humans, 1997-2015: a systematic review of individual case data. *Lancet Infect. Dis.* 16 E108–E118. 10.1016/S1473-3099(16)00153-5 27211899 PMC4933299

[B37] Lamb R. A. Lai C. J. (1980). Sequence of interrupted and uninterrupted mRNAs and cloned DNA coding for the two overlapping nonstructural proteins of influenza virus. *Cell* 21 475–485. 10.1016/0092-8674(80)90484-5 7407920

[B38] Lessler J. Reich N. G. Cummings D. A. Nair H. P. Jordan H. T. Thompson N. (2009). Outbreak of 2009 pandemic influenza A (H1N1) at a New York City school. *N. Engl. J. Med.* 361 2628–2636. 10.1056/NEJMoa0906089 20042754

[B39] Li G. Ju J. Weyand C. M. Goronzy J. J. (2015). Age-Associated failure to adjust type I IFN receptor signaling thresholds after T cell activation. *J. Immunol.* 195 865–874. 10.4049/jimmunol.1402389 26091718 PMC4506866

[B40] Livak K. J. Schmittgen T. D. (2001). Analysis of relative gene expression data using real-time quantitative PCR and the 2(-Delta Delta C(T)) method. *Methods* 25 402–408. 10.1006/meth.2001.1262 11846609

[B41] Loo Y. M. Gale M. Jr. (2007). Influenza: fatal immunity and the 1918 virus. *Nature* 445 267–268. 10.1038/445267a 17230179

[B42] Ludwig S. Schultz U. Mandler J. Fitch W. M. Scholtissek C. (1991). Phylogenetic relationship of the nonstructural (NS) genes of influenza A viruses. *Virology* 183 566–577. 10.1016/0042-6822(91)90985-K 1830182

[B43] Lv J. Wang D. Hua Y. H. Pei S. J. Wang J. Hu W. W. (2014). Pulmonary immune responses to 2009 pandemic influenza A (H1N1) virus in mice. *BMC Infect. Dis.* 14:197. 10.1186/1471-2334-14-197 24725777 PMC4002205

[B44] Ma J. Shen H. Liu Q. Bawa B. Qi W. Duff M. (2015). Pathogenicity and transmissibility of novel reassortant H3N2 influenza viruses with 2009 pandemic H1N1 genes in pigs. *J. Virol.* 89 2831–2841. 10.1128/JVI.03355-14 25540372 PMC4325708

[B45] Ma W. Brenner D. Wang Z. Dauber B. Ehrhardt C. Hogner K. (2010). The NS segment of an H5N1 highly pathogenic avian influenza virus (HPAIV) is sufficient to alter replication efficiency, cell tropism, and host range of an H7N1 HPAIV. *J. Virol.* 84 2122–2133. 10.1128/JVI.01668-09 20007264 PMC2812369

[B46] Marc D. (2014). Influenza virus non-structural protein NS1: interferon antagonism and beyond. *J. Gen. Virol.* 95 2594–2611. 10.1099/vir.0.069542-0 25182164

[B47] Marcus P. I. Rojek J. M. Sekellick M. J. (2005). Interferon induction and/or production and its suppression by influenza A viruses. *J. Virol.* 79 2880–2890. 10.1128/JVI.79.5.2880-2890.2005 15709007 PMC548469

[B48] Mathieu C. Moreno V. Retamal P. Gonzalez A. Rivera A. Fuller J. (2010). Pandemic (H1N1) 2009 in breeding turkeys, Valparaiso, Chile. *Emerg. Infect. Dis.* 16 709–711. 10.3201/eid1604.091402 20350395 PMC3321954

[B49] Melen K. Kinnunen L. Fagerlund R. Ikonen N. Twu K. Y. Krug R. M. (2007). Nuclear and nucleolar targeting of influenza A virus NS1 protein: striking differences between different virus subtypes. *J. Virol.* 81 5995–6006. 10.1128/JVI.01714-06 17376915 PMC1900311

[B50] Meunier I. Embury-Hyatt C. Stebner S. Gray M. Bastien N. Li Y. (2012). Virulence differences of closely related pandemic 2009 H1N1 isolates correlate with increased inflammatory responses in ferrets. *Virology* 422 125–131. 10.1016/j.virol.2011.10.018 22074911

[B51] Morens D. M. Fauci A. S. (2007). The 1918 influenza pandemic: insights for the 21st century. *J. Infect. Dis.* 195 1018–1028. 10.1086/511989 17330793

[B52] Mostafa A. Kanrai P. Petersen H. Ibrahim S. Rautenschlein S. Pleschka S. (2015). Efficient generation of recombinant influenza A viruses employing a new approach to overcome the genetic instability of HA segments. *PLoS One* 10:e0116917. 10.1371/journal.pone.0116917 25615576 PMC4304806

[B53] Mostafa A. Kanrai P. Ziebuhr J. Pleschka S. (2013). Improved dual promotor-driven reverse genetics system for influenza viruses. *J. Virol. Methods* 193 603–610. 10.1016/j.jviromet.2013.07.021 23886561

[B54] Mostafa A. Kanrai P. Ziebuhr J. Pleschka S. (2016). The PB1 segment of an influenza A virus H1N1 2009pdm isolate enhances the replication efficiency of specific influenza vaccine strains in cell culture and embryonated eggs. *J. Gen. Virol.* 97 620–631. 10.1099/jgv.0.000390 26743314

[B55] Muhlbauer D. Dzieciolowski J. Hardt M. Hocke A. Schierhorn K. L. Mostafa A. (2015). Influenza virus-induced caspase-dependent enlargement of nuclear pores promotes nuclear export of viral ribonucleoprotein complexes. *J. Virol.* 89 6009–6021. 10.1128/JVI.03531-14 25810542 PMC4442457

[B56] Mukherjee S. Vipat V. C. Mishra A. C. Pawar S. D. Chakrabarti A. K. (2011). Pandemic (H1N1) 2009 influenza virus induces weaker host immune responses in vitro: a possible mechanism of high transmissibility. *Virol. J.* 8:140. 10.1186/1743-422X-8-140 21439068 PMC3076257

[B57] Nelson M. I. Stratton J. Killian M. L. Janas-Martindale A. Vincent A. L. (2015). Continual re-introduction of human pandemic H1N1 influenza A viruses into US Swine, 2009-2014. *J. Virol.* 1:JVI.00459-15. 10.1128/JVI.00459-15 PMC4474294 25833052

[B58] Neumann G. Noda T. Kawaoka Y. (2009). Emergence and pandemic potential of swine-origin H1N1 influenza virus. *Nature* 459 931–939. 10.1038/nature08157 19525932 PMC2873852

[B59] Osterlund P. Veckman V. Siren J. Klucher K. M. Hiscott J. Matikainen S. (2005). Gene expression and antiviral activity of alpha/beta interferons and interleukin-29 in virus-infected human myeloid dendritic cells. *J. Virol.* 79 9608–9617. 10.1128/JVI.79.15.9608-9617.2005 16014923 PMC1181545

[B60] Pantin-Jackwood M. Wasilenko J. L. Spackman E. Suarez D. L. Swayne D. E. (2010). Susceptibility of turkeys to pandemic-H1N1 virus by reproductive tract insemination. *Virol. J.* 7:27. 10.1186/1743-422X-7-27 20128914 PMC2830961

[B61] Pappas C. Viswanathan K. Chandrasekaran A. Raman R. Katz J. M. Sasisekharan R. (2010). Receptor specificity and transmission of H2N2 subtype viruses isolated from the pandemic of 1957. *PLoS One* 5:e11158. 10.1371/journal.pone.0011158 20574518 PMC2888575

[B62] Perrone L. A. Plowden J. K. Garcia-Sastre A. Katz J. M. Tumpey T. M. (2008). H5N1 and 1918 pandemic influenza virus infection results in early and excessive infiltration of macrophages and neutrophils in the lungs of mice. *PLoS Pathog.* 4:e1000115. 10.1371/journal.ppat.1000115 18670648 PMC2483250

[B63] Petersen H. Matrosovich M. Pleschka S. Rautenschlein S. (2012). Replication and adaptive mutations of low pathogenic avian influenza viruses in tracheal organ cultures of different avian species. *PLoS One* 7:e42260. 10.1371/journal.pone.0042260 22912693 PMC3418272

[B64] Petersen H. Wang Z. Lenz E. Pleschka S. Rautenschlein S. (2013). Reassortment of NS segments modifies highly pathogenic avian influenza virus interaction with avian hosts and host cells. *J. Virol.* 87 5362–5371. 10.1128/JVI.02969-12 23468508 PMC3648139

[B65] Pleschka S. Wolff T. Ehrhardt C. Hobom G. Planz O. Rapp U. R. (2001). Influenza virus propagation is impaired by inhibition of the Raf/MEK/ERK signalling cascade. *Nat. Cell Biol.* 3 301–305. 10.1038/35060098 11231581

[B66] Powell F. L. Rothwell L. Clarkson M. J. Kaiser P. (2009). The turkey, compared to the chicken, fails to mount an effective early immune response to *Histomonas meleagridis* in the gut. *Parasite Immunol.* 31 312–327. 10.1111/j.1365-3024.2009.01113.x 19493211

[B67] Ranaware P. B. Mishra A. Vijayakumar P. Gandhale P. N. Kumar H. Kulkarni D. D. (2016). Genome wide host gene expression analysis in chicken lungs infected with avian influenza viruses. *PLoS One* 11:e0153671. 10.1371/journal.pone.0153671 27071061 PMC4829244

[B68] Reid S. M. Cox W. J. Ceeraz V. Sutton D. Essen S. C. Howard W. A. (2012). First reported detection of influenza A (H1N1)pdm09 in turkeys in the United Kingdom. *Avian Dis.* 56 1062–1067. 10.1637/10178-041012-Reg.1 23402137

[B69] Sato Y. Yoshioka K. Suzuki C. Awashima S. Hosaka Y. Yewdell J. (2003). Localization of influenza virus proteins to nuclear dot 10 structures in influenza virus-infected cells. *Virology* 310 29–40. 10.1016/S0042-6822(03)00104-1 12788628

[B70] Shelton H. Smith M. Hartgroves L. Stilwell P. Roberts K. Johnson B. (2012). An influenza reassortant with polymerase of pH1N1 and NS gene of H3N2 influenza A virus is attenuated *in vivo*. *J. Gen. Virol.* 93 998–1006. 10.1099/vir.0.039701-0 22323532 PMC3541804

[B71] Shi W. Shi Y. Wu Y. Liu D. Gao G. F. (2013). Origin and molecular characterization of the human-infecting H6N1 influenza virus in Taiwan. *Protein Cell* 4 846–853. 10.1007/s13238-013-3083-0 24136722 PMC4875453

[B72] Short K. R. Brooks A. G. Reading P. C. Londrigan S. L. (2012). The fate of influenza A virus after infection of human macrophages and dendritic cells. *J. Gen. Virol.* 93 2315–2325. 10.1099/vir.0.045021-0 22894921

[B73] Short K. R. Richard M. Verhagen J. H. Van Riel D. Schrauwen E. J. Van Den Brand J. M. (2015). One health, multiple challenges: the inter-species transmission of influenza A virus. *One Health* 1 1–13. 10.1016/j.onehlt.2015.03.001 26309905 PMC4542011

[B74] Sid H. Hartmann S. Petersen H. Ryll M. Rautenschlein S. (2016). *Mycoplasma gallisepticum* modifies the pathogenesis of influenza A virus in the avian tracheal epithelium. *Int. J. Med. Microbiol.* 306 174–186. 10.1016/j.ijmm.2016.04.001 27079856

[B75] Sun H. Kong W. Liu L. Qu Y. Li C. Shen Y. (2015). The infection of turkeys and chickens by reassortants derived from pandemic H1N1 2009 and avian H9N2 influenza viruses. *Sci. Rep.* 5:10130. 10.1038/srep10130 26030097 PMC4603695

[B76] Swayne D. E. Pantin-Jackwood M. Kapczynski D. Spackman E. Suarez D. L. (2009). Susceptibility of poultry to pandemic (H1N1) 2009 virus. *Emerg. Infect. Dis.* 15 2061–2063. 10.3201/eid1512.091060 19961704 PMC3044551

[B77] Szretter K. J. Gangappa S. Lu X. Smith C. Shieh W. J. Zaki S. R. (2007). Role of host cytokine responses in the pathogenesis of avian H5N1 influenza viruses in mice. *J. Virol.* 81 2736–2744. 10.1128/JVI.02336-06 17182684 PMC1866007

[B78] Tisoncik J. R. Korth M. J. Simmons C. P. Farrar J. Martin T. R. Katze M. G. (2012). Into the eye of the cytokine storm. *Microbiol. Mol. Biol. Rev.* 76 16–32. 10.1128/MMBR.05015-11 22390970 PMC3294426

[B79] Tu J. Guo J. Zhang A. Zhang W. Zhao Z. Zhou H. (2011). Effects of the C-terminal truncation in NS1 protein of the 2009 pandemic H1N1 influenza virus on host gene expression. *PLoS One* 6:e26175. 10.1371/journal.pone.0026175 22022552 PMC3192165

[B80] Tumpey T. M. Garcia-Sastre A. Taubenberger J. K. Palese P. Swayne D. E. Pantin-Jackwood M. J. (2005). Pathogenicity of influenza viruses with genes from the 1918 pandemic virus: functional roles of alveolar macrophages and neutrophils in limiting virus replication and mortality in mice. *J. Virol.* 79 14933–14944. 10.1128/JVI.79.23.14933-14944.2005 16282492 PMC1287592

[B81] Turnbull M. L. Wise H. M. Nicol M. Q. Smith N. Dunfee R. L. Beard P. M. (2016). Role of the B allele of influenza A virus segment 8 in setting mammalian host range and pathogenicity. *J. Virol.* 90 9263–9284. 10.1128/JVI.01205-16 27489273 PMC5044859

[B82] Twu K. Y. Noah D. L. Rao P. Kuo R. L. Krug R. M. (2006). The CPSCPSF30 binding site on the NS1A protein of influenza A virus is a potential antiviral target. *J. Virol.* 80 3957–3965. 10.1128/JVI.80.8.3957-3965.2006 16571812 PMC1440456

[B83] Wang X. Tan J. Zoueva O. Zhao J. Ye Z. Hewlett I. (2014). Novel pandemic influenza A (H1N1) virus infection modulates apoptotic pathways that impact its replication in A549 cells. *Microbes Infect.* 16 178–186. 10.1016/j.micinf.2013.11.003 24262752

[B84] Wang Z. Robb N. C. Lenz E. Wolff T. Fodor E. Pleschka S. (2010). NS reassortment of an H7-type highly pathogenic avian influenza virus affects its propagation by altering the regulation of viral RNA production and antiviral host response. *J. Virol.* 84 11323–11335. 10.1128/JVI.01034-10 20739516 PMC2953208

[B85] Watanabe T. Zhong G. Russell C. A. Nakajima N. Hatta M. Hanson A. (2014). Circulating avian influenza viruses closely related to the 1918 virus have pandemic potential. *Cell Host Microbe* 15 692–705. 10.1016/j.chom.2014.05.006 24922572 PMC4205238

[B86] Watanabe Y. Ibrahim M. S. Ellakany H. F. Kawashita N. Mizuike R. Hiramatsu H. (2011). Acquisition of human-type receptor binding specificity by new H5N1 influenza virus sublineages during their emergence in birds in Egypt. *PLoS Pathog.* 7:e1002068. 10.1371/journal.ppat.1002068 21637809 PMC3102706

[B87] Watson S. J. Langat P. Reid S. M. Lam T. T. Y. Cotten M. Kelly M. (2015). Molecular epidemiology and evolution of influenza viruses circulating within European Swine between 2009 and 2013. *J. Virol.* 89 9920–9931. 10.1128/JVI.00840-15 26202246 PMC4577897

[B88] Wright S. M. Kawaoka Y. Sharp G. B. Senne D. A. Webster R. G. (1992). Interspecies transmission and reassortment of influenza-A viruses in pigs and Turkeys in the United-States. *Am. J. Epidemiol.* 136 488–497. 10.1093/oxfordjournals.aje.a116522 1415168

[B89] Yang N. Hong X. Yang P. Ju X. Wang Y. Tang J. (2011). The 2009 pandemic A/Wenshan/01/2009 H1N1 induces apoptotic cell death in human airway epithelial cells. *J. Mol. Cell Biol.* 3 221–229. 10.1093/jmcb/mjr017 21816972

[B90] Yang P. Deng J. Li C. Zhang P. Xing L. Li Z. (2012). Characterization of the 2009 pandemic A/Beijing/501/2009 H1N1 influenza strain in human airway epithelial cells and ferrets. *PLoS One* 7:e46184. 10.1371/journal.pone.0046184 23049974 PMC3458874

[B91] Yoneyama M. Suhara W. Fukuhara Y. Fukuda M. Nishida E. Fujita T. (1998). Direct triggering of the type I interferon system by virus infection: activation of a transcription factor complex containing IRF-3 and CBP/p300. *EMBO J.* 17 1087–1095. 10.1093/emboj/17.4.1087 9463386 PMC1170457

[B92] Zaffuto K. M. Estevez C. N. Afonso C. L. (2008). Primary chicken tracheal cell culture system for the study of infection with avian respiratory viruses. *Avian Pathol.* 37 25–31. 10.1080/03079450701774850 18202946

[B93] Zhang C. Yang Y. Zhou X. Yang Z. Liu X. Cao Z. (2011). The NS1 protein of influenza A virus interacts with heat shock protein Hsp90 in human alveolar basal epithelial cells: implication for virus-induced apoptosis. *Virol. J.* 8:181. 10.1186/1743-422X-8-181 21501532 PMC3098181

[B94] Zhu H. Lam T. T. Smith D. K. Guan Y. (2016). Emergence and development of H7N9 influenza viruses in China. *Curr. Opin. Virol.* 16 106–113. 10.1016/j.coviro.2016.01.020 26922715

