# Supplementary material for: NS Segment of a 1918 Influenza A Virus-Descendent Enhances Replication of H1N1pdm09 and Virus-Induced Cellular Immune Response in Mammalian and Avian Systems
Source: Front Microbiol. 2018 Mar 22;9:526. doi: 10.3389/fmicb.2018.00526 (PMC5874506; doi:10.3389/fmicb.2018.00526)
Supplement: Supplementary file 1 [file Presentation_1.PDF]

Supplementary Material

NS Segment Reassortment Can Increase Growth Kinetics and Pathogenicity of the 2009 Pandemic H1N1 Influenza A Virus *In Vitro* and *In Vivo*

Henning Petersen, Ahmed Mostafa, Mohamed A Tantawy, Azeem A. Iqbal, Donata Hoffmann, Aravind Tallam, Balachandar Selvakumar, Frank Pessler, Martin Beer, Silke Rautenschlein and Stephan Pleschka*

*** Correspondence:** Prof. Dr. Stephan Pleschka (Email: stephan.pleschka@viro.med.uni-giessen.de)

1 Supplementary Figures

1.1 Supplementary Figure 1

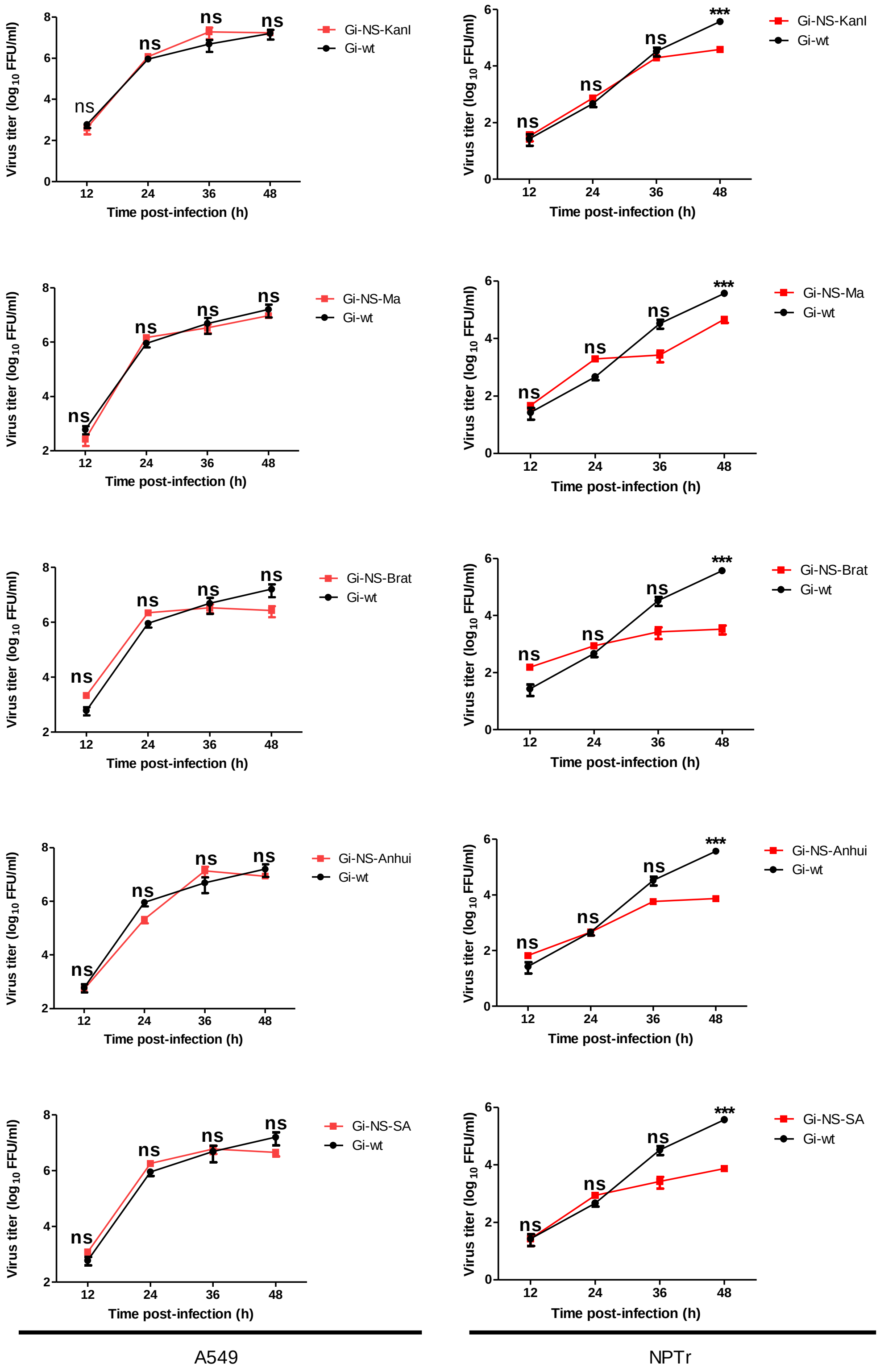

Supplementary Figure 1. Growth kinetics of different Gi-NS-reassortants and Gi-wt in human A549 and swine NPTr cells. Cells were infected with different Gi-NS-reassortants and recombinant Gi-wt at an MOI of 0.001 FFU/cell. Supernatants were collected at 12, 24, 36, and 48 h p.i.. Collected samples were then titrated using foci assay (FFU/ml) in MDCK cells. The titers were calculated for three independent experiments; error bars indicate the standard deviation (SD). Statistical analysis was performed using "repeated measures ANOVA", followed by "Bonferroni post hoc" test. (*) $p < 0.05$; (**) $p < 0.001$; (***) $p < 0.0001$; ns, non-significant. Error bars represent standard deviation (SD).

1.2 Supplementary Figure 2

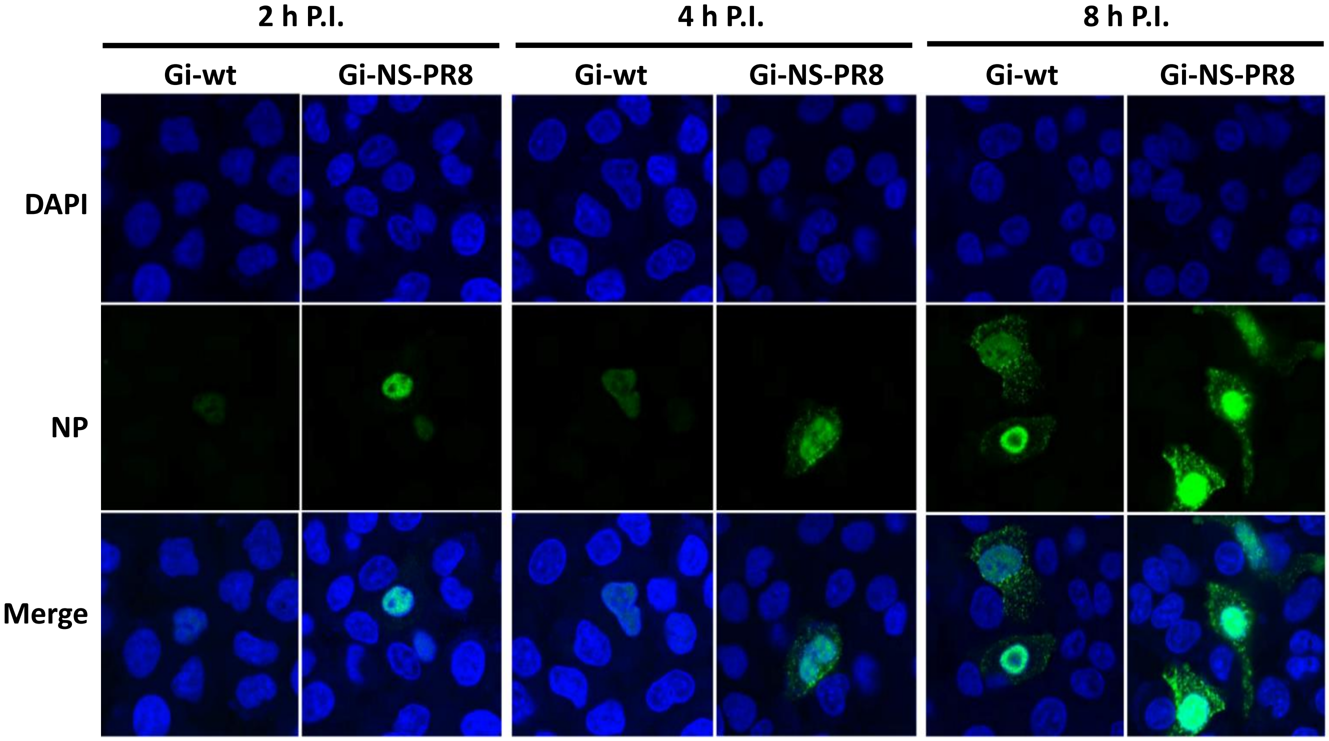

Supplementary Figure 2. vRNP export of recombinant Gi-wt and reassortant Gi-NS-PR8. A549 cells were infected with recombinant viruses (MOI=1). After 2, 4, 8 h p.i., A549 cells were processed for immunofluorescence. The viral NP protein (green) indicates the RNP localisation; nuclei were stained with DAPI (blue).

1.3 Supplementary Figure 3

(A)

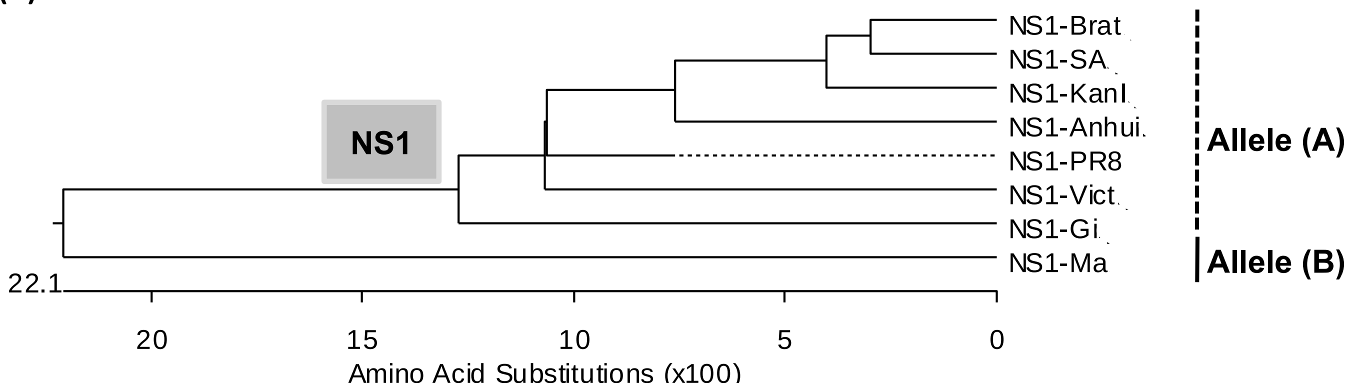

(B)

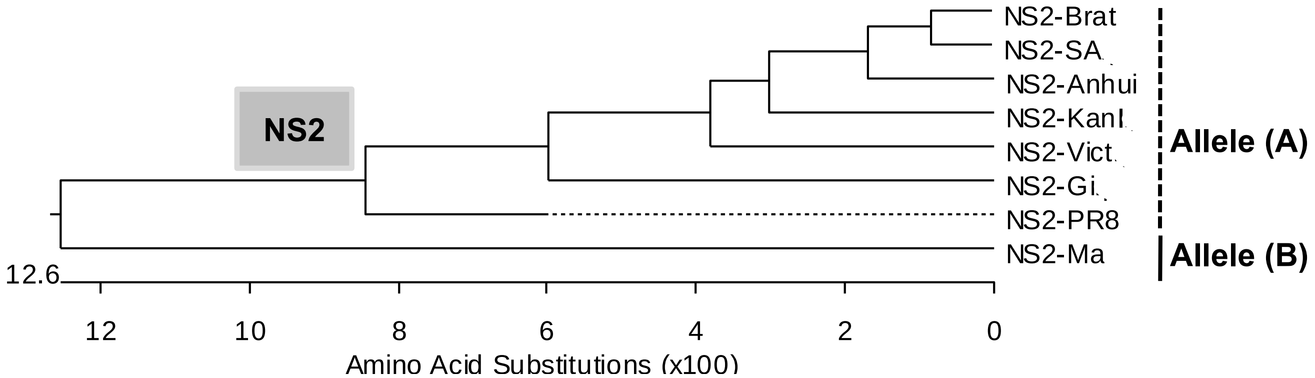

(C)

```
                    190       200        210        220        230
NS1-Brat.pro        NTVRVSETLQRFAWRSSNEDRRPPLPPKQKRKMARTIESEV              230 aa
NS1-Gi.pro          NGNTVRVSENIQRFAWRNCDENGRPSLPPEQK                       219 aa
NS1-KanI.pro        NDNTVRVTETIQRFAWRSSDEDGRLPLPPNQKRKMARTIESEV            225 aa
NS1-Ma.pro          NDNSIRASENIQRFAWGIRDENGGPPLPPKQKRYMARRVESEV            230 aa
NS1-PR8.pro         NDNTVRVSETLQRFAWRSSNENGRPPLTPKQKREMAGTIRSEV            230 aa
NS1-SA.pro          NDNTVRVSETLQRFAWRGSNEDGRPPLPPKQKRKMARTIESEV            230 aa
NS1-Vict.pro        NDNTVRVSKTLQRFAWGSSNENGGPPLTPKQKRKMARTARSKVRRDKMAD     237 aa
NS1-Anhui.pro       NDNTVRVSETLQRFAWRSSDEDGRSPLSTK                         217 aa
```

Supplementary Figure 3. Phylogenetic analysis of the different NS1 and NS2 proteins encoded by the different NS segments. (A) The NS1 aa sequences were aligned by ClustalW program in the DNA Star software to generate the phylogenetic tree (Lasergene, WI). (B) Phylogenetic tree of the studied NS2 proteins. (C) Truncations and prolongation of disordered tails of different NS1 used.
